# *N*-Acylated amino acid methyl esters from marine *Roseobacter* group bacteria

**DOI:** 10.3762/bjoc.14.276

**Published:** 2018-12-03

**Authors:** Hilke Bruns, Lisa Ziesche, Nargis Khakin Taniwal, Laura Wolter, Thorsten Brinkhoff, Jennifer Herrmann, Rolf Müller, Stefan Schulz

**Affiliations:** 1 Institute of Organic Chemistry, Technische Universität Braunschweig, Hagenring 30, 38106 Braunschweig, Germany; 2 Institute for Chemistry and Biology of the Marine Environment, University of Oldenburg, Carl-von-Ossietzky-Straße 9–11, 26111 Oldenburg, Germany; 3 Helmholtz Institute for Pharmaceutical Research Saarland (HIPS), Helmholtz Center for Infection Research (HZI), Saarland University, Campus E8.1, 66123 Saarbrücken, Germany

**Keywords:** amino acid derivatives, 2-aminobutyric acid, homoserine lactones, natural products, quorum sensing

## Abstract

Bacteria of the *Roseobacter* group (Rhodobacteraceae) are important members of many marine ecosystems. Similar to other Gram-negative bacteria many roseobacters produce *N*-acylhomoserine lactones (AHLs) for communication by quorum sensing systems. AHLs regulate different traits like cell differentiation or antibiotic production. Related *N*-acylalanine methyl esters (NAMEs) have been reported as well, but so far only from *Roseovarius tolerans* EL-164. While screening various roseobacters isolated from macroalgae we encountered four strains, *Roseovarius* sp. D12_1.68, *Loktanella* sp. F13, F14 and D3 that produced new derivatives and analogs of NAMEs, namely *N*-acyl-2-aminobutyric acid methyl esters (NABME), *N*-acylglycine methyl esters (NAGME), *N*-acylvaline methyl esters (NAVME), as well as for the first time a methyl-branched NAME, *N*-(13-methyltetradecanoyl)alanine methyl ester. These compounds were detected by GC–MS analysis, and structural proposals were derived from the mass spectra and by derivatization. Verification of compound structures was performed by synthesis. NABMEs, NAVMEs and NAGMEs are produced in low amounts only, making mass spectrometry the method of choice for their detection. The analysis of both EI and ESI mass spectra revealed fragmentation patterns helpful for the detection of similar compounds derived from other amino acids. Some of these compounds showed antimicrobial activity. The structural similarity of *N*-acylated amino acid methyl esters and similar lipophilicity to AHLs might indicate a yet unknown function as signalling compounds in the ecology of these bacteria, although their singular occurrence is in strong contrast to the common occurrence of AHLs. Obviously the structural motif is not restricted to *Roseovarius* spp. and occurs also in other genera.

## Introduction

The identification and structural elucidation of naturally occurring compounds traditionally requires isolation and NMR investigation as key method to detect novel compounds and new structural classes. Although the advent of NMR spectrometers with high frequencies and cryoprobes with small diameters enables experiments to be performed in the μg scale with pure compound, the isolation of the pure material from complex samples as well as the access to the expensive equipment still pose a considerable challenge to find new compounds. The ongoing quest for new structures also increasingly addresses minor components, requiring larger amounts of the producing organism, not always readily accessible, to isolate a targeted compound [1–3].

An alternative methodology can avoid the laborious isolation procedure. Direct analysis by mass-spectrometric methods of natural materials, e.g., extracts, may give enough information to infer the structure of an unknown compound that is finally proven by synthesis and comparison with natural material. The use of GC/EI-MS is especially advantageous because such mass spectra often reveal key structural features. Furthermore, the availability of large cross-platform databases useful for dereplication allows focussing on new compounds.

We are interested in natural compounds from *Roseobacter* group bacteria, an abundant class of marine bacteria occurring in diverse habitats with a broad metabolic potential [4–7]. Especially attached-living roseobacters produce diverse secondary metabolites, e.g., *N*-acylhomoserine lactones (AHLs) that the bacteria use for communication by quorum sensing [8–10]. AHLs are extensively investigated because of the broad knowledge on their biosynthesis, the underlying gene organization, as well as their function in many bacteria [11–13]. In the *Roseobacter* group, AHLs are involved, e.g., in antibiotic production [9] or cell differentiation [10]. Although many other bacterial signalling compounds must exist, only few of them have been characterized so far [14–18]. Such signalling compounds as well as many other unknown metabolites often occur in small amounts, which renders trace detecting methods like GC/MS a suitable approach for their detection and structure elucidation, provided their polarity falls into the analytical window of the method.

**Figure 1:**
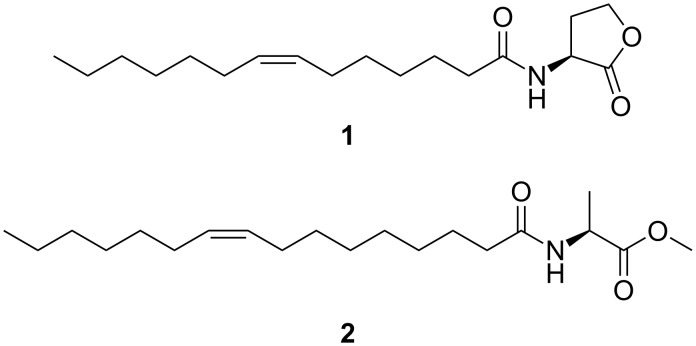
*N*-Acylhomoserine lactones **1** (Z7-C14:1-AHL) and *N*-acylalanine methyl esters **2** (Z9-C16:1-NAME) occurring in *Roseobacter* group bacteria.

A wide variety of AHLs, e.g., widespread (*Z*)-*N*-(tetradec-7-enoyl)homoserine lactone (**1**, Z7-C-14:1-AHL, Figure 1), have been identified in roseobacters by these methods [19–22]. A related group of compounds occurring in *Roseovarius* only, are *N*-acylalanine methyl esters (NAMEs), e.g., (*Z*)-*N*-(hexadec-9-enoyl)alanine methyl ester (**2**, Z9-C16:1-NAME), the major NAME produced by *Roseovarius tolerans* EL 164 [23]. Although NAMEs are structurally similar to AHLs by an acyl chain linked to an amino acid derivative via an amide bond, they do not activate AHL receptors in roseobacters [21]. Instead, they show moderate antialgal activity [21]. In contrast to AHLs, the acyl chain can also be terminally oxidized [24]. During our analyses of different *Roseobacter* isolates, we encountered several compounds, which mass spectra show similarities to known NAMEs. These compounds proved to be either new NAMEs or constitute new classes of acylated amino acid methyl esters, derived from valine (NAVME), glycine (NAGME), or 2-aminobutyric acid (NABME). The identification of these compounds will be discussed based on the outlined approach including GC/MS analysis, interpretation of mass spectra, and verification by synthesis.

## Results and Discussion

The secondary metabolites released by liquid cultures of various roseobacters were collected by extraction via Amberlite XAD-16 resin and analysed by GC/MS. Four of these strains, *Roseovarius* sp. D12_1.68 and *Loktanella* sp. F13, F14 and D3, contained low amounts of compounds with similar mass spectra to those of NAMEs [23].

### *Roseovarius* sp. D12_1.68

**Figure 2:**
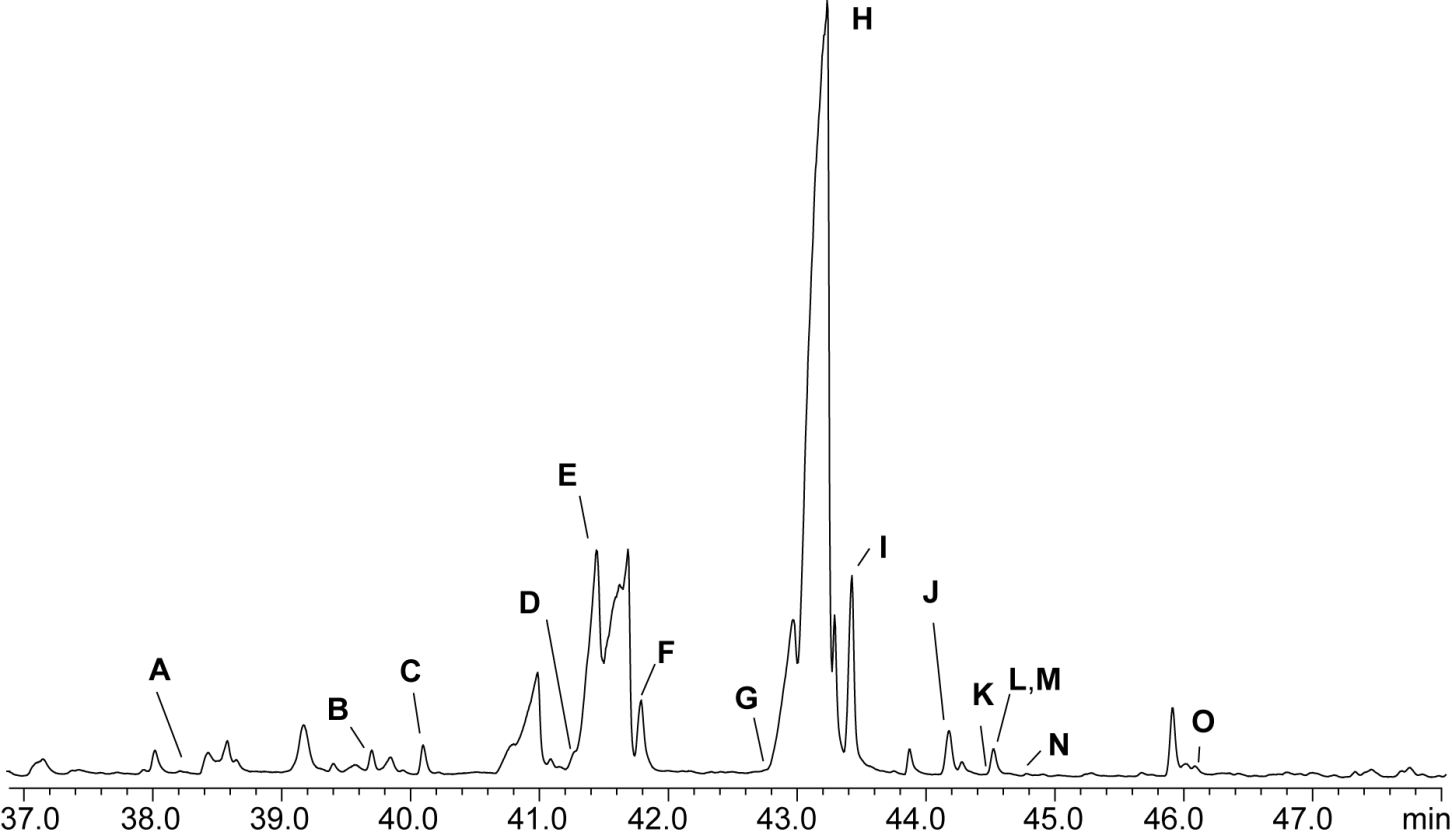
Total ion chromatogram (TIC) of an XAD extract of *Roseovarius* sp. D12_1.68. AHLs, NAMEs and related compounds are assigned by bold letters (Table 1).

The investigation of an extract by GC/MS (Figure 2) revealed the presence of several NAMEs and AHLs due to their characteristic ions at *m*/*z* 104, 145, and 158 and *m*/*z* 102, 143, and 156, respectively [21,23]. Some of these compounds, **E** and **L** in Figure 2, were readily identified by their mass spectra and gas chromatographic retention indices *I*_nat_ as known AHLs, containing saturated C_12_ and C_14_ acyl chains (Table 1).

**Table 1:** Composition of the extracellular metabolites of *Roseovarius* sp. D12_1.68. [M]^+^, *m/z*: molecular and characteristic ions in EI mass spectrum. *I*_nat_: gas chromatographic retention index of natural compounds. HRMS: HR-mass spectral data of [M + H]^+^ obtained by HPLC/HR–ESI^+^–MS.

peak	compound	[M]^+^	*m*/*z*	*I*_nat_	HRMS [M + H]^+^

**A**	C13:0-NAME	299	104, 145, 158	2181	
**B**	C14:1-NAME	311	104, 145, 158	2265	C_18_H_34_NO_3_
**C**	C14:0-NAME	313	104, 145, 158	2289	C_18_H_36_NO_3_
**D**	*iso*-C15:0-NAME	327	104, 145, 158	2354	C_19_H_38_NO_3_
**E**	C12:0-AHL	283	102, 143, 156	2367	
**F**	C15:0-NAME	327	104, 145, 158	2392	C_19_H_38_NO_3_
**G**	C16:1-NAME	339	104, 145, 158	2457	C_20_H_38_NO_3_
**H**	Z9-C16:1-NAME	339	104, 145, 158	2473	C_20_H_38_NO_3_
**I**	C16:0-NAME	341	104, 145, 158	2497	C_20_H_40_NO_3_
**J**	9-C16:1-NABME	353	118, 159, 172	2548	C_21_H_40_NO_3_
**K**	C16:0-NABME	355	118, 159, 172	2569	C_21_H_42_NO_3_
**L**	C14:0-AHL	311	102, 143, 156	2570	
**M**	9-C17:1-NAME	353	104, 145, 158	2570	C_21_H_40_NO_3_
**N**	9-C16:1-NAVME	367	132, 173, 181	2588	C_22_H_42_NO_3_
**O**	11-C18:1-NAME	367	104, 145, 158	2677	C_22_H_42_NO_3_

Similarly, compounds **C**, **F**, **H**, **I**, and **M** were identified as the already known C14:0-, C15:0-, 9-C16:1-, C16:0-, and 9-C17:1-NAMEs. Compounds **A**, **B**, and **O** proved to be not previously reported C13:0-, C14:1- and C18:1-NAMEs, assignable by their mass spectra and gas chromatographic retention indices. These conclusions were supported by HRMS data obtained by HPLC/MS (Table 1). Localization of the double bond was established via DMDS derivatization as described previously [23]. Due to the low amounts no double bond position could be established for C14:1-NAME, while the double bond of C18:1 NAME was located at C-11. Similar as in AHLs, the double bond location in unsaturated NAMEs seems to be fixed at the ω-7 position [8,25].

**Scheme 1:**
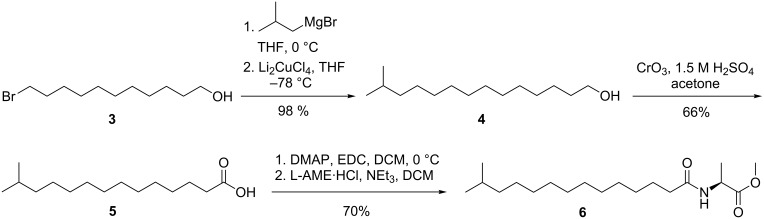
Synthesis of *iso*-C15:0-NAME (**6**). DMAP: 4-dimethylaminopyridine, EDC: 1-ethyl-3-(3-dimethylaminopropyl)carbodiimide hydrochloride, L-AME: 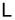-alanine methyl ester.

Compound **D** (C_19_H_37_NO_3_) showed a mass spectrum identical to C15:0-NAME, albeit the retention index deviated by 38 units. A methyl branch at *iso*- or *anteiso-*position seemed likely. Therefore, the theoretical retention index *I*_c_ were calculated for methyl branched C15:0-NAMEs using an empirical model established in our work group [26] that had successfully been used for the detection of methyl branched AHLs [27]. The retention indices were calculated using the formula


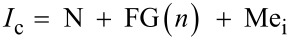


with N indicating the number of *n* carbons in the chain times hundred, FG as the functional group increment depending on and Me_i_ as an increment for the methyl branching in different positions. The increments for Me_i_ are known [26]. The functional group increment was calculated to be 836 + 4 · *n* using the retention indices of C14:0, C15:0, and C16:0-NAME. Therefore, the calculated retention index for *iso*-C15:0 and *anteiso*-C15:0-NAME are *I*_c_ = 2352 and *I*_c_ = 2365, respectively, while all other methyl branch locations had a lower value. The close similarity of *I*_nat_ = 2354 and *I*_c_ = 2352 suggested the methyl branch to be located in the *iso*-position. Consequently, *iso*-C15:0-NAME (**6**) was synthesized as shown in Scheme 1 to verify the structural proposal.

**Figure 3:**
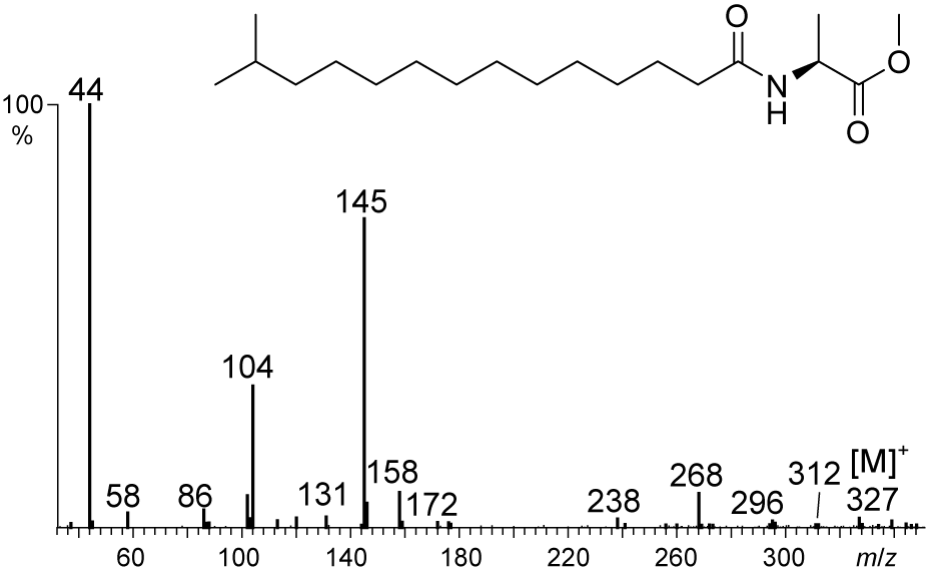
Mass spectrum of natural compound **D**, *N*-(13-methyltetradecanoyl)alanine methyl ester (*iso*-C15:0-NAME, **6**).

11-Bromoundecan-1-ol (**3**) was converted into the alcohol 13-methyltetradecan-1-ol (**4**) with isobutylmagnesium bromide under Li_2_CuCl_4_ catalysis according to Mori et al. [28]. After Jones oxidation, 13-methyltetradecanoic acid (**5**) was coupled with 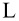-alanine methyl ester hydrochloride to deliver the desired product **6**. The mass spectra and retention indices of the natural and synthetic samples were identical, proving the proposed structure (Figure 3). This compound is the first natural NAME featuring a methyl branched acyl chain.

Compound **G** showed a mass spectrum identical to C16:1-NAME and a retention index with *I* = 2457, eluting earlier than compound **H**, Z9-C16:1-NAME with *I* = 2473. The low amount of the material produced excluded further structural characterization of the compound that could either be methyl-branched in the acyl chain or might show a different double bond position or configuration.

**Figure 4:**
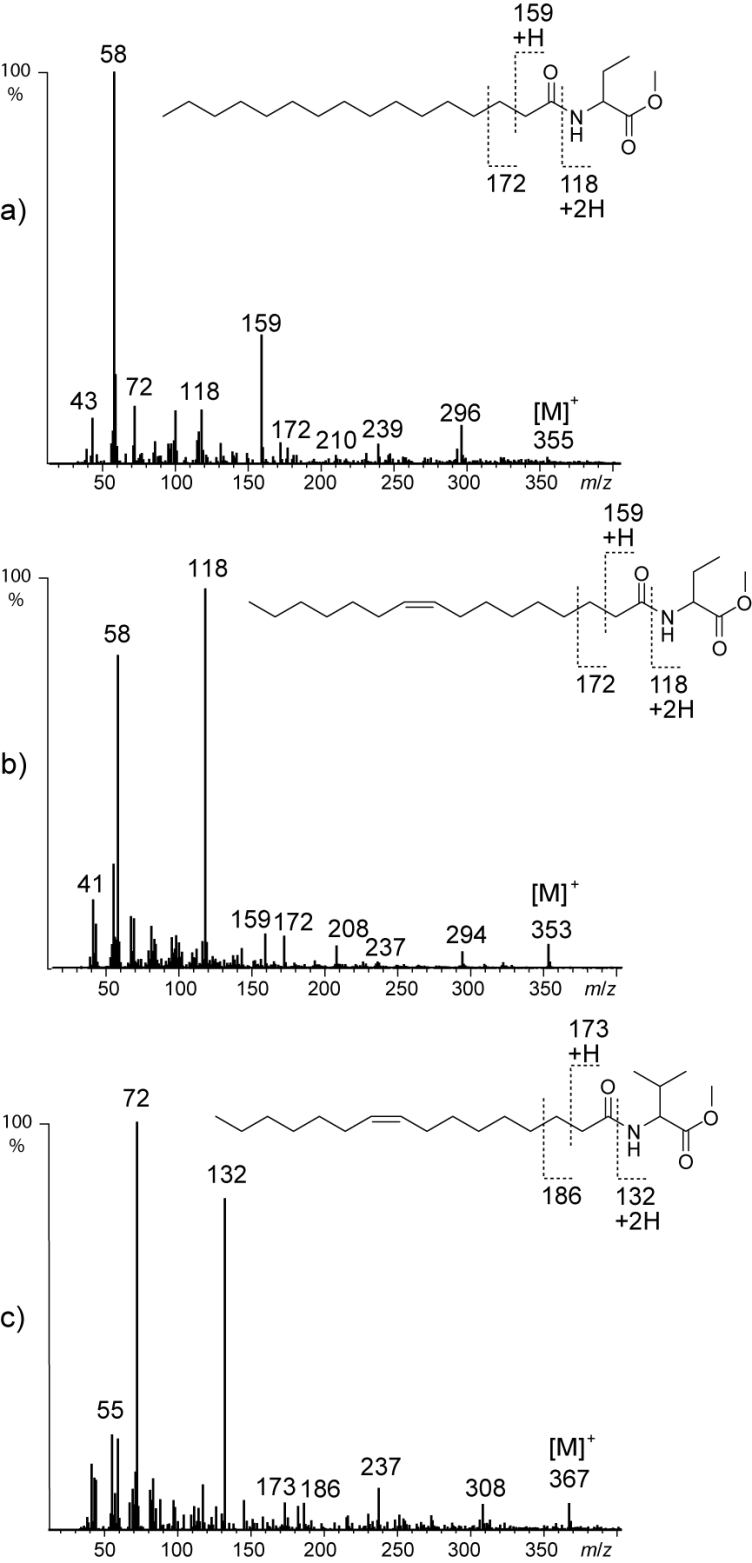
Mass spectra of natural compounds a) **K** (**7**, C16:0-NABME), b) **J** (**8**, C16:1-NABME), and c) **N** (**9**, C16:1-NAVME).

The remaining three compounds **J**, **K**, and **N** with the molecular composition C_21_H_42_NO_3_, C_21_H_40_NO_3_, and C_22_H_42_NO_3_ determined by HRMS showed related mass spectra with a characteristic mass shift compared to NAMEs. Ions *m*/*z* 44, 104, 145, and 158 where shifted, however, to *m*/*z* 58, 118, 159, and 172 in the spectra of **J** and **K** (Figure 4a,b).

**Scheme 2:**
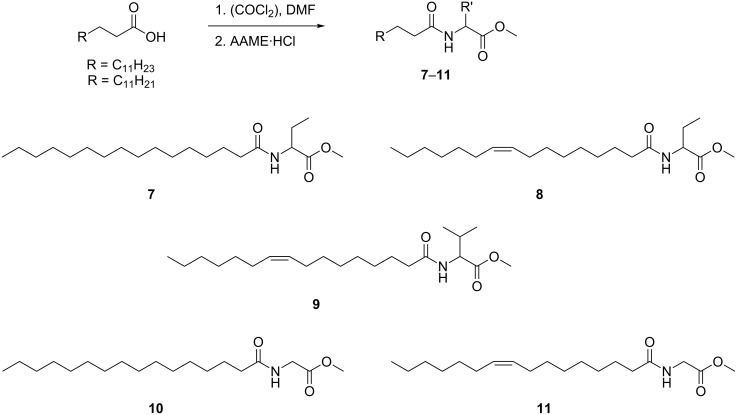
Synthesis of *N*-acylated amino acid methyl esters **7**–**11**. AAME: amino acid methyl ester.

These ions can be explained by an additional CH_2_ group in the alanine part, leading to a 2-aminobutyric acid fragment in these compounds. The later eluting compound **K** with a molecular ion at *m/z* 355 was therefore proposed to be *N*-(hexadecanoyl)-2-aminobutyric acid methyl ester (**7**), while the earlier eluting **J** with *m/z* 353 compound was likely *N*-[(*Z*)-hexadec-9-enoyl]-2-aminobutyric acid methyl ester (**8**). The double bond position was determined by DMDS derivatization. The structures of both **K** and **J** were verified by synthesis according to Scheme 2. Palmitoleic acid was synthesized in g-scale by standard procedures as shown in the Supporting Information File 1, Scheme S1. This acid and palmitic acid were converted into the respective chlorides and standard acylation delivered 2-aminobutyric acid derivatives **7** and **8** (Scheme 2) that proved to be identical with the natural products. The absolute configuration of the amino acid could not be determined due to the low amount of material present. Nevertheless, because NAMEs showed the common 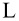-configuration [23], this configuration also seems likely for the other amino acid derivatives reported here. We suggest the term NABME (*N*-acylated 2-aminobutyric acid methyl esters) for the new compounds that can thus be assigned as C16:0-NABME (**7**) and Z9-C16:1-NABME (**8**).

Compound **N** showed a mass spectrum (Figure 4c) featuring characteristic ions at *m*/*z* 72, 132, and 173, indicating a further carbon in the amino acid part. Together with the molecular ion at *m/z* 367 (C_22_H_41_NO_3_) the data indicate the presence of valine in the compound. DMDS derivatization proved the unknown compound to also carry a double bond at C-9 of the alkenoyl chain. The proposed structure *N-*[(*Z*)-hexadec-9-enoyl]valine methyl ester (**9**) was proven by synthesis starting from valine methyl ester as described (Scheme 2). The gas chromatographic retention indices of the synthetic material and the natural compound matched perfectly. We propose to call the *N*-acylated valine methyl ester **9** Z9-C16:1-NAVME.

The extract of *Roseovarius* sp. D12_1.68 was also investigated by HPLC/ESI^+^–MS to detect more polar compounds compared to GC. The NAMEs, NABMEs and NAVMEs reported here were detected by MS^2^ analyses based on their characteristic fragmentation (see below). The only oxygenated derivative present was 16OH-C16:1-NAME, which has been described before from *Roseovarius tolerans* EL-164 [24].

### *Loktanella* sp. D3, F13 and F14

**Figure 5:**
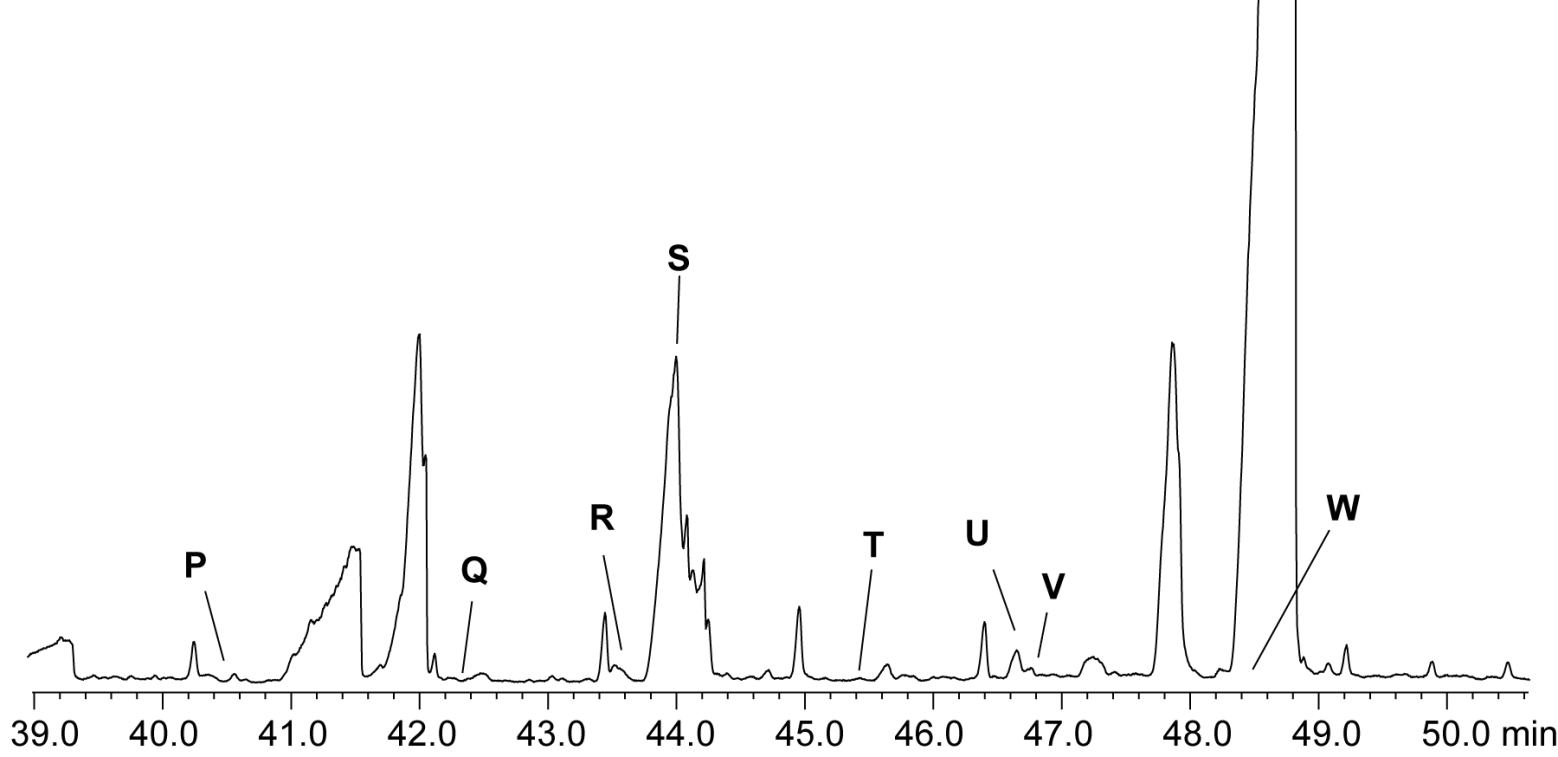
TIC of an XAD extract of *Loktanella* sp. F14. Compound **S** is a minor component within the broad peak.

**Figure 6:**
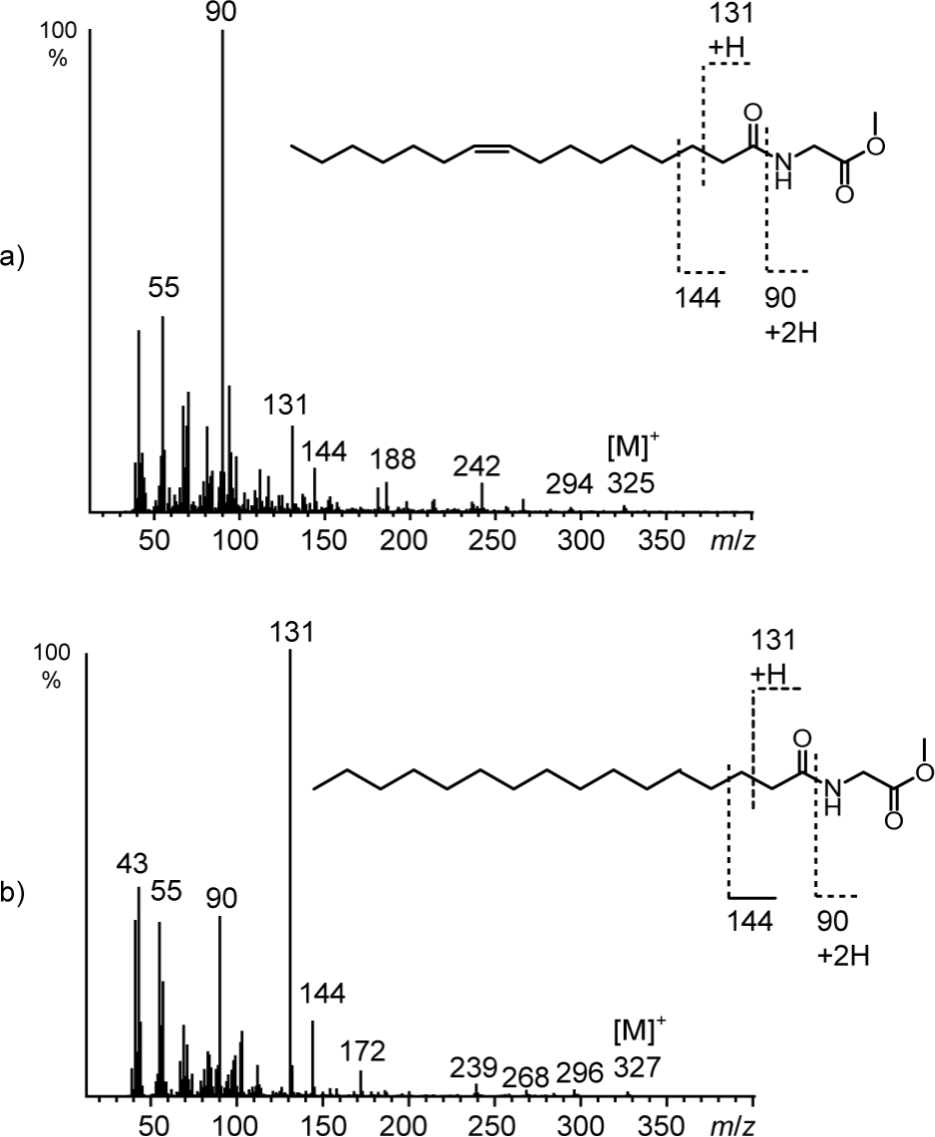
Mass spectra of natural compounds a) **R** (**11**, C16:1-NAGME) and b) **S** (**10**, C16:0-NAGME).

Investigation of extracts of the three isolates F14, F13, and D3 by GC–MS indicated the presence of compounds whose mass spectra were again similar to those of NAMEs (Figure 5). The spectra show characteristic ions at *m*/*z* 90, 131, and 144 (Figure 6), a loss of one methylene group compared to ions *m*/*z* 104, 145, and 158 of NAMEs. The lack of an analogous ion to *m/z* 44 (*m/z* 30 is outside the mass range of the spectrometer used) pointed this time to glycine as the core amino acid.

Molecular formulas were obtained via HPLC/HRMS measurements and supported the *N*-acylglycine methyl ester (NAGME) structure proposed for these compounds.

Saturated and unsaturated NAGMEs can be distinguished by the intensity of the ions *m*/*z* 90 and 131. Similar to NAMEs saturated NAGMEs show a high intensity of *m*/*z* 131 whereas unsaturated NAGMEs show higher intensity of *m*/*z* 90 (Figure 6). The low amounts available did not allow to determine the position of the double bond in unsaturated NAGMEs. Nevertheless, the predominance of the (*Z*)-9-hexadecenoyl side chain in all NAME family compounds suggested compound **R** to be *N*-[(*Z*)-hexadec-9-enoyl]glycine methyl ester (**11**, Z9-C16:1-NAGME), while **S** is its saturated analogue. Therefore, both compounds were synthesized as described before from glycine methyl ester and the respective acid (Scheme 2) and their identity confirmed. The other components **P**, **Q** and **T**–**W** were also NAGMEs.

Their chain length was established using EI mass spectra and the gas chromatographic retention indices *I_nat_* of the compounds (Table 2). Overall, six saturated and unsaturated compounds with a chain length between C_14_ and C_19_ were detected. The roughly 100 retention index units between the members of this homologous series indicated that the acyl chains are unbranched. Additionally, two unsaturated glycine derivatives were present, 9-C16:1-NAGME (**11**) and C18:1-NAGME (Table 2).

**Table 2:** NAGMEs produced by *Loktanella* related isolates F14, F13 and D3. *I_nat_*: gas chromatographic retention index on a HP-5 phase; [M]^+^: molecular mass; *m*/*z*: characteristic ions in EI mass spectra.

peak	compound	*I_nat_*	[M]*^+^*	*m*/*z*	*Loktanella* isolate		
					
					F14	F13	D3

**P**	C14:0-NAGME	2312	299	131 > 90	x	x	
**Q**	C15:0-NAGME	2415	313	131 > 90	x	x	
**R**	C16:1-NAGME	2495	325	90 < 131	x	x	
**S**	C16:0-NAGME	2515	327	131 > 90	x	x	x
**T**	C17:0-NAGME	2618	341	131 > 90		x	
**U**	C18:1-NAGME	2702	353	90 < 131	x	x	
**V**	C18:0-NAGME	2720	355	131 > 90	x	x	
**W**	C19:0-NAGME	2832	369	131 > 90	x		

Analysis by HPLC/MS revealed no additional NAGME. Furthermore, no NAMEs, NABMEs, or NAVMEs were observed in the three strains.

### Mass spectrometry

**Figure 7:**
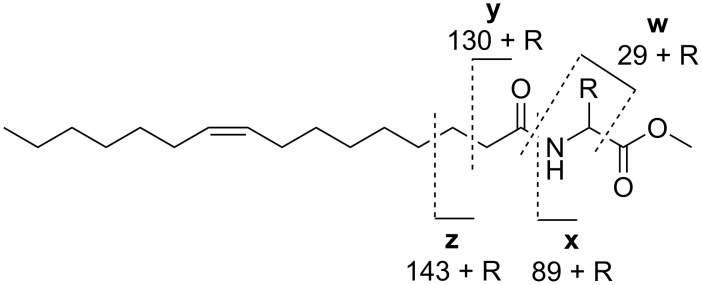
EI mass spectrometric fragmentation of *N*-acylated amino acid derivatives. The ions **w**–**z** allow identification of the respective amino acid residue.

**Figure 8:**
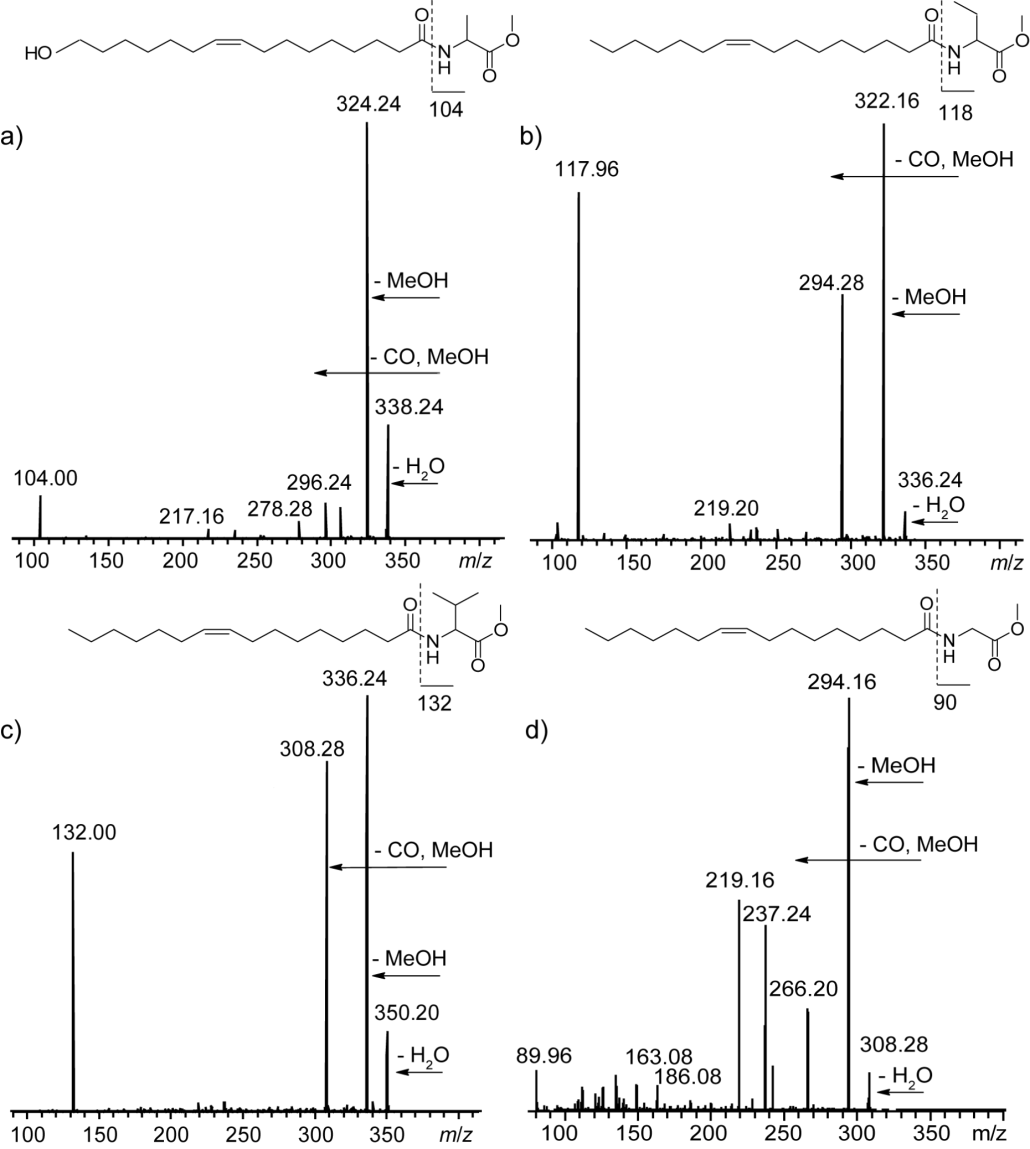
MS^2^ spectra in ESI positive mode of a) Z9-16OH-C16:1-NAME with [M + H − H_2_O]^+^ 356, b) Z9-C16:1-NABME (**8**) with [M + H]^+^ 354, c) Z9-C16:1-NAVME (**9**) with [M + H]^+^ 368, and d) Z9-C16:1-NAGME (**11**) with [M + H]^+^ 326.

The analysis of the mass spectra of NAMEs, NABMEs, NAVMEs, and NAGMEs revealed the typical fragmentation of *N*-acylated amino acid methyl esters under both EI (Figure 7) and ESI ionization (Figure 8). Detailed structural information can be obtained by EI-MS. A dominant peak in the mass spectrum is the McLafferty rearrangement ion **y**, if the acyl chain is saturated. Together with prominent ion **w** [NH–CH_2_–R]^+^, often the base peak, and **x** it defines the amino acid, while **z** is usually of low abundance. Formation of x requires transfer of two H atoms to this fragment. In compounds carrying an unsaturated acyl chain the intensity of **y** is reduced, and **x** increases in intensity. In addition to these features, the molecular ion is visible, as is the loss of the carbomethoxy group [M − 59]^+^.

The MS^2^ spectrum of the [M + H]^+^-ion obtained in ESI positive mode shows loss of water, an intense ion due to loss of methanol, and loss of the carbomethoxy group [24]. Often the amino acid ion can also be observed. These features indicate the presence of an amino acid methyl ester.

### Biological activity

Several of the synthesized compounds were tested in a broader screening program for their antimicrobial activity (Table 3). While the valine derivative **9** was virtually inactive, the other compounds showed some activity. Glycine compound **11** showed good activity against the Gram-positive bacteria *Bacillus subtilis*, *Staphylococcus aureus*, and *Micrococcus luteus*, and against the filamentous fungus *Mucor hiemalis*. In addition, **11** displayed moderate active on *Mycobacterium smegmatis* and the efflux-deficient *Escherichia coli* TolC strain. The 2-aminobutyric acid derivative **7** was active against *M. hiemalis*, *M. luteus* and *S. aureus,* while the unsaturated analogue **8** was mainly active against *E. coli* TolC, and it was the only compound that showed moderate cytotoxicity on a human cancer cell line.

**Table 3:** Antimicrobial activity (minimum inhibitory concentration, MIC, in µg/mL) and cytotoxicity (minimum inhibitory concentration, MIC, in µg/mL) of selected NAVME, NABME and NAGME derivatives. Minimal inhibitory concentration and IC_50_ values for cytotoxicity in μg/mL.

strain	**11**	**9**	**8**	**7**

*Chromobacterium violaceum* DSM-30191	>128	>128	nd	>128
*Escherichia coli* DSM-1116	>128	>128	nd	>128
*Escherichia coli* (TolC-deficient)	128	>128	16	>128
*Pseudomonas aeruginosa* PA14	>128	>128	nd	>128
*Bacillus subtilis* DSM-10	4–8	>128	>128	>128
*Micrococcus luteus* DSM-1790	4	>128	64–128	16
*Staphylococcus aureus* Newman	16	>128	nd	8–16
*Mycobacterium smegmatis* mc^2^155	64	>128	nd	>128
*Mucor hiemalis* DSM-2656	8–16	>128	nd	32
*Pichia anomala* DSM-6766	>128	>128	nd	>128
*Candida albicans* DSM-1665	>128	>128	nd	>128

cytotoxicity				

HCT-116 (human colon carcinoma)	>67	>67	15.8	>67

The close similarity of NAMEs, NAVMEs, and NAGMEs to AHLs might indicate a function as signalling compounds, although experiments with NAMEs and AHL reporter assays did not reveal any activity on the LuxR type receptors for AHLs in bacteria [23–24]. Other potential functions are antimicrobial or cytotoxic activity of the tested derivatives. *Roseovarius* sp. D12_1.68 showed antimicrobial activity against a Rhodobacteraceae sp. TL and antialgal activity against *Skeletonema costatum* while *Loktanella* sp. D3 showed no antagonistic activity [29]. This observation fits well with the detection of antimicrobial or cytotoxic NAVME **9** and NABMEs **7** and **8** only in *Roseovarius* sp. D12_1.68.

Bacteria are known to produce acylated amino acids, although the number reported so far is small, including tyrosine, tryptophan, arginine, or phenylalanine [30–32]. They all carry long chain saturated or unsaturated acyl chains similar to those reported here. Recently, derivatives of the hydrophobic amino acids valine, leucine and isoleucine were also reported [33]. These compounds are produced by a family of acyl amino acid synthases structurally related to AHL synthases, further suggesting a function as bacterial signalling compounds [34]. In contrast to the reported compounds carrying a free acid group, all derivatives reported here are native methyl esters because no methanol was used during sample preparation

## Conclusion

We have identified here new classes of acylated amino acid derivatives including previously unknown glycine and 2-aminobutyric acid derived compounds. The combination of GC/MS, HPLC/MS, retention indices and synthesis proved to be especially suited to structurally identify minor components of complex extracellular metabolite mixtures. The reported compounds are specific for *Roseobacter* group bacteria of the genera *Roseovarius* and *Loktanella*, in contrast to broadly distributed AHLs. Although their function as signalling compounds is not proven, the occurrence of 2-aminobutyric acid might indicate some similarity to homoserine in AHLs because both are non-proteinogenic amino acids. This similarity might also be functional, because the structures of NAMEs, NABMEs, NAGMEs and NAVMEs are similar to other bacterial signalling compounds, often carrying a lipophilic side chain and a medium polar core structure [8,16]. Nevertheless, the ecological function of NAMEs and its derivatives could also be antagonistic activity against concurrent biofilm microorganisms, suggested by the bioactivity of some of the compounds as observed in this study and the antimicrobial and antialgal activity of the producing organism [29].

## Supporting Information

File 1 Experimental synthetic procedures, biological tests and NMR spectra.
