# Supplementary material for: N-Acylated amino acid methyl esters from marine Roseobacter group bacteria
Source: Beilstein J Org Chem. 2018 Dec 3;14:2964–73. doi: 10.3762/bjoc.14.276 (PMC6296433; doi:10.3762/bjoc.14.276)
Supplement: File 1 — Experimental synthetic procedures, biological tests and NMR spectra. [file Beilstein_J_Org_Chem-14-2964-s001.pdf]

BEILSTEIN JOURNAL OF ORGANIC CHEMISTRY

Supporting Information

for

N-Acylated amino acid methyl esters from marine *Roseobacter* group bacteria

Hilke Bruns, Lisa Ziesche, Nargis Khakin Taniwal, Laura Wolter, Thorsten Brinkhoff, Jennifer Herrmann, Rolf Müller and Stefan Schulz

Beilstein J. Org. Chem. **2018,** *14,* 2964–2973. doi:10.3762/bjoc.14.276

Experimental synthetic procedures, biological tests and NMR spectra

License and Terms: This is a supporting information file under the terms of the Creative Commons Attribution License (http://creativecommons.org/licenses/by/4.0). Please note that the reuse, redistribution and reproduction in particular requires that the authors and source are credited.
The license is subject to the *Beilstein Journal of Organic Chemistry* terms and conditions: (https://www.beilstein-journals.org/bjoc)

Contents

Experimental	S3
Synthetic procedures	S6
NMR spectra	S20
References	S26

Experimental

General. IR Spectra: Bruker Tensor 27 ATR spectrometer. UV Spectra: Varian Cary 100 Bio spectrometer. NMR Spectra: Bruker DRX-400 (400 MHz), AV III-400 (400 MHz), or AV II-600 (600 MHz) spectrometers; referenced to TMS (δ 0.00 ppm) for ^{1}H NMR and $CDCl_3$ (δ 77.01 ppm) for ^{13}C NMR, chemical shifts are in ppm, coupling constants *J* in Hz. LC–MS: Only LC–MS grade eluents were used. LC–MS data were acquired on a Thermo Fischer Accela equipped with pump, autosampler, and PDA detector, connected to a LTQ XL from Thermo Fischer. The measurements were carried out in ESI positive mode. The temperature of the ion source was 40 °C and the capillary temperature was 275 °C. The sheath gas flow was 15 mL/min and auxiliary gas 10 mL/min. MS^2 analyses were carried out in CID mode with a normalized collision energy of 35%. The activation Q was 0.250 and Activation time 30 ms. Spectra were evaluated with Thermo Xcalibur 2.2 software. GC–MS: HP6890 GC system connected to a HP5973 Mass Selective Detector fitted with a BPX-5 fused silica cap. column (25 m, 0.22 mm i.d., 0.25 mm film, SGE Inc., Melbourne, Australia); conditions: inlet pressure 97.0 kPa, He 45.5 mL/min; injection volume 1 µL; injector 250 °C, transfer line 300 °C, electron energy 70 eV. The GC was programmed as follows: 50 °C (5 min isotherm), increasing at 10 °C/min to 320 °C, and operated in split mode (35:1), carrier gas (He) 1.2 mL/min. GC–MS analyses of XAD extracts and of the synthesized compounds: Agilent GC 7890A system connected to a 5975C mass-selective detector (Agilent) fitted with a HP-5 MS fused silica capillary column (30 m, 0.25 mm i. d., 0.22 mm film, Hewlett-Packard, Wilmington, USA), conditions: inlet pressure 67.5 kPa, He 24.2 mL/min, injection volume 1 µL, injector 250 °C, transfer line 300 °C, electron energy 70 eV. The GC was programmed as follows: 50 °C (5 min isothermal), increasing at 5 °C/min to

320 °C, and operated in splitless mode, carrier gas (He) 1.2 mL/min. Gas chromatographic retention indices (*I*) were determined from a homologous series of *n*-alkanes (C_8–C_{33}).

Chemicals were purchased from Sigma Aldrich Chemie GmbH (Steinheim, Germany) or from Acros Organics (Geel, Belgium), and used without further purification. Solvents were purified by distillation and dried according to standard procedures. Moisture- and/or oxygen-sensitive reactions were carried out under N_2 in vacuum-heated flasks with dried solvents. Amberlite XAD-16 was purified before by washing with 30 mL methanol and 3 × 30 mL distilled water. TLC: 0.20 mm Macherey-Nagel silica gel plates (Polygram SIL G/UV254). Column chromatography (CC): Merck silica gel 60 (0.040–0.063 mm) using standard flash chromatographic methods.

Strains, culture conditions, and extraction. *Roseovarius* sp. D12_1.68 (KM268065) and *Loktanella* sp. F13 (KJ786460) and D3 (KC731427) were isolated during the analysis of the epibacterial community associated with *Fucus spiralis* from a tidal flat area of the southern North Sea in Germany, collected from a rocky site in summer 2010. *Loktanella* sp. F14 was isolated from a marine green alga Ulva sp. on the same date and location. In a similar manner as described in [S1], precultures were routinely grown in marine broth medium (MB, Carl Roth, Karlsruhe, Germany) in Erlenmeyer flasks at 28 °C on a rotary shaker at 160 rpm. Erlenmeyer flasks (500 mL) containing 100 mL of MB were inoculated with 2% preculture, and 2% of Amberlite XAD-16 was added. After growth of culture (3–5 days), the resin was filtered off and extracted with 3 × 10 mL of CH_2Cl_2/H_2O 2:1 (ν/ν) mixture. The two phases were separated, the organic phase was dried ($MgSO_4$), and the solvent was evaporated under reduced pressure. The extract was concentrated at 60 °C under N_2

to a volume of ca. 500 µL. For HPLC analysis, 400 µL of an extract was evaporated to dryness and dissolved in 300 µL of MeCN.

LC–MS of XAD extracts. The dried extract was dissolved in MeCN/H_2O 1:1 and filtered prior to analysis. An Agilent Eclipse Plus C18 column (3.5 µm, 2.1 × 150 mm) was used with a flow of 250 µL/min and an injection volume of 10 µL. The method used was 92.5% H_2O (B), 2.5% MeCN (C) and 5% MeCN containing 2% formic acid (D) from 0–1.5 min, 2.5% B, 92.5% C, 5% D from 8–15 min and reequilibration from 17–22 min to 92.5% B, 2.5% C and 5% D.

Bioassays. All compounds were dissolved in DMSO and their minimum inhibitory concentrations (MIC) were tested in standard microbroth dilution assays as described earlier [S2]. Cultures of *Bacillus subtilis* DSM-10, *Staphylococcus aureus* Newman, *Micrococcus luteus* DSM-1790, *Escherichia coli* DSM-1116, TolC-deficient *E. coli, Chromobacterium violaceum* DSM-30191, *Pseudomonas aeruginosa* PA14, *Mycobacterium smegmatis* mc^2155, *Mucor hiemalis* DSM-2656, *Pichia anomala* DSM-6766, *Candida albicans* DSM-1665 in mid-log phase were diluted to achieve a final inoculum of ca. 5×10^5–5×10^6 cfu/mL in Mueller-Hinton broth (1.75% casein hydrolysate, 0.2% beef infusion, 0.15% starch; pH 7.4; used for all bacteria, except *M. smegmatis*), M7H9 medium (Difco™ Middlebrook 7H9 broth supplemented with BBL™ Middlebrook ADC enrichment and 2 mL/L glycerol; used for *M. smegmatis*) or Myc medium (1% phytone peptone, 1% glucose, 50 mM HEPES, pH 7.0; used for yeasts and fungi). Serial dilutions of samples were prepared from DMSO stock solutions in sterile 96-well plates, the cell suspension was added, and microorganisms were grown for 16–48 h at their optimal growth temperature at either

30 °C or 37 °C. Given MIC values are the lowest concentration of antibiotic at which there was no visible growth.

Synthetic procedures

13-Methyltetradecan-1-ol (4)

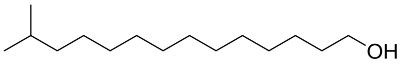

4

The reaction was carried out under dry conditions. A small layer of dry THF was placed on magnesium chips (0.90 g, 36.82 mmol, 3.7 equiv), a small portion of pure 1-bromo-2-methylpropane (4.01 mL, 5.05 g, 36.82 mmol, 3.7 equiv) was added and the mixture slightly heated [S3]. When the ether started to boil the remaining 1-bromo-2-methylpropane, dissolved in dry THF (15 mL), was added slowly. The mixture started to boil and after 30 min of reflux the solution was allowed to cool to room temperature. In another flask 1-bromoundecan-1-ol (**3**, 2.50 g, 9.95 mmol, 1 equiv) in dry THF (20 mL) was cooled with ice and the previously prepared isobutylmagnesium bromide solution was added slowly. The mixture was cooled to −78 °C and Li_2CuCl_4 solution (0.1 M in THF, 1.49 mL, 0.015 equiv) was added. The mixture was allowed to warm to room temperature and stirred for 5 h. The mixture was again cooled with an ice bath and hydrolyzed with 2 M HCl until the precipitate dissolved. After separation of the phases and extraction of the water phase with diethyl ether (3 × 60 mL) the combined organic layers were washed with saturated NaCl solution (50 mL) and distilled water (30 mL). The organic layers were dried with $MgSO_4$, the solvent was evaporated under reduced pressure and the residue was subjected to column chromatography which yielded compound **4** (2.22 g, 9.72 mmol, 98%) as a white solid.

GC (HP-5MS): *I* = 1739;

TLC [Pentane/diethyl ether (1:1)]: R_f = 0.30;

^{1}H-NMR ($CDCl_3$, 400 MHz) δ = 3.64 (t, *J* = 6.6 Hz, CH_2OH, 2H), 1.60-1.53 (m, 2H), 1.53 (non, *J* = 6.7 Hz, 1 H), 1.36-1.22 (m, 18H), 1.17 -1.12 (m, 2H), 0.86 (d, *J* = 6.6 Hz, 6 H) ppm; ^{13}C-NMR ($CDCl_3$, 100 MHz): δ = 63.1 (CH_2OH), 39.1 (CH_2), 32.8 (CH_2), 29.9 (CH_2), 29.7 (CH_2), 29.7 (CH_2), 29.7 (CH_2), 29.6 (CH_2), 29.6 (CH_2), 29.4 (CH_2), 28.0 (CH), 27.4 (CH_2), 25.7 (CH_2), 22.6 (2 x CH_3) ppm; MS (70 eV, EI): *m*/*z* (%) = 228 (<1, $[M]^+$), 210 (5, $[M-H_2O]^+$), 182 (26), 154 (31), 125 (24), 111 (56), 97 (77), 83 (90), 69 (100), 55 (87), 43 (66); IR (ATR): 1/λ = 3346 (w, br), 2921 (s), 2852 (s), 1466 (m), 1366 (w), 1054 (m), 720 (m), 605 (w), 580 (w) cm^{-1}

13-Methyltetradecanoic acid (5)

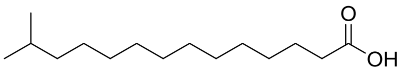

5

Alcohol **4** (1.20 g, 5.25 mmol, 1 equiv) was dissolved in acetone (70 mL, HPLC grade) and cooled with ice. A solution of CrO_3 (2.10 g, 21.02 mmol, 4 equiv) in H_2SO_4 (1.5 M, 31.5 mL) was prepared and slowly added to the alcohol solution so that the temperature did not rose above 5–10°C. The solution was allowed to warm to room temperature, washed with sat. NaCl solution (100 mL) and extracted with diethyl ether (3 × 50 mL). The combined organic phases were washed with sat. NaCl solution, dried with $MgSO_4$ and the solvent was evaporated under reduced pressure [S4,S5]. After column chromatography compound **5** (0.84 g, 3.47 mmol, 66%) was obtained as a white solid.

TLC [Pentane/diethyl ether containing 1 % acetic acid (6:1)]: R_f = 0.29;

^{1}H-NMR ($CDCl_3$, 400 MHz) δ = 2.35 (t, J = 7.5 Hz, 2 H, CH_2COOH), 1.63 (q, J = 7.5 Hz, 2 H, CH_2CH_2COOH), 1.51 (non, J = 6.6 Hz, 1 H), 1.35-1.21 (m, 16H), 1.17-1.12 (m, 2H), 0.86 (d, J = 6.6 Hz, 6 H) ppm; ^{13}C-NMR ($CDCl_3$, 100 MHz): δ = 180.1 (C=O), 39.1 (CH_2), 34.1 (CH_2), 29.9 (CH_2), 29.7 (CH_2), 29.7 (CH_2), 29.6 (CH_2), 29.4 (CH_2), 29.2 (CH_2), 29.1 (CH_2), 28.0 (CH), 27.4 (CH_2), 24.7 (CH_2), 22.7 (2 x CH_3) ppm; IR (ATR): $1/\lambda$ = 2954 (m), 2916 (s), 2849 (s), 1694 (s), 1470 (m), 1430 (m), 1408 (m), 1381 (w), 1362 (w), 1304 (m), 1286 (m), 1263 (m), 1237 (m), 1213 (m), 1191 (m), 1098 (w), 934 (m, br), 773 (w), 719 (m), 683 (m), 544 (m) cm^{-1}; UV/VIS (CH_2Cl_2): λ_{max} (log ε) = 222 (2.05), 224 (2.25), 230 (2.25) nm.

_N_-(13-Methyltetradecanoyl)alanine methyl ester (6, iso-15:0-NAME)

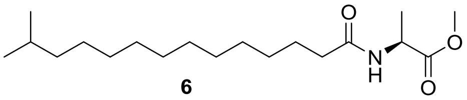

6

In a similar manner as described in [S1], acid **5** (0.20 g, 0.83 mmol, 1 equiv) was dissolved in abs. CH_2Cl_2 (10 mL) under a N_2 atmosphere. After addition of 4-(*N*,*N*-dimethylamino)pyridine (DMAP, 0.10 g, 0.83 mmol, 1 equiv), 1-ethyl-3-(3-dimethylaminopropyl)carbodiimide (EDC, 0.19 mL, 1.07 mmol, 1.3 equiv) was added at 0 °C and the solution was stirred for 1 h at 0 °C. The resulting mixture was allowed to warm to room temperature and stirred for an additional hour. A solution of L-alanine methyl ester hydrochloride (0.15 g, 1.07 mmol, 1.3 equiv) in abs. CH_2Cl_2 (10 mL) was prepared and triethylamine (0.15 mL, 1.07 mmol, 1.3 equiv) was added. This solution was added dropwise to the solution of **5** and stirred over night at room temperature. The solution was washed with 1 M HCl (10 mL), $NaHCO_3$ (10 mL) and H_2O (10 mL), the phases were separated and the organic phase dried with $MgSO_4$

[S6,S7]. Product **6** (0.19 g, 0.58 mmol, 70%) was obtained after column chromatography as a white solid (pentane/ethyl acetate 2:1).

GC (HP-5MS): *I* = 2353;

TLC [Pentane/diethyl ether (2:1)]: R_f = 0.39;

^{1}H-NMR ($CDCl_3$, 300 MHz): δ = 6.03 (d, *J* = 6.6 Hz, 1 H, NH), 4.61 (q, *J* = 7.3 Hz, 1 H), 3.75 (s, 3 H), 2.21 (t, *J* = 7.8 Hz, 2 H), 1.65-1.58 (m, 2H, C$H_2$$CH_2$C=O), 1.51 (non, 1H, *J* = 6.5 Hz, CH), 1.40 (d, 3H, *J* = 7.1 Hz, $CHCH_3$); 1.29-1.20 (m, 16H, 8 x CH_2), 1.18-1.12 (m, 2H, CH_2), 0.86 (d, 6H, *J* = 6.6 Hz, 3 x CH_2) ppm; ^{13}C-NMR ($CDCl_3$, 75 MHz): δ = 173.7 (NHC=O), 172.6 (C=O), 52.4 (OCH_3), 47.8 (CH), 39.0 (CH_2C=O), 36.6 (CH_2), 29.9 (CH_2), 29.7 (CH_2), 29.6 (CH_2), 29.6 (CH_2), 29.5 (CH_2), 29.3 (CH_2), 29.2 (CH_2), 27.9 (CH), 27.4 (CH_2), 25.6 (CH_2), 22.6 (2 x CH_3), 18.6 (CH_2CH_3) ppm; MS (70 eV, EI): *m*/*z* (%) = 327 (3, $[M]^+$), 296 (2), 268 (12), 158 (12), 145 (93), 104 (28), 86 (7), 69 (9), 55 (17), 44 (100).

Methyl (*Z*)-*N*-hexadec-9-enoylvalinate (9)

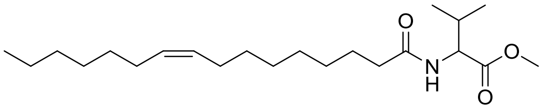

9

Dimethylformamide (1 drop) was added to a solution of palmitoleic acid (**S7**) (0.100 g, 0.393 mmol, 1 equiv) in CH_2Cl_2 (2 mL). Oxalyl chloride (0.074 g, 0.586 mmol, 1.5 equiv) in CH_2Cl_2 (1 mL) was added dropwise to the solution at 0 °C. The resulting solution was left to stir at room temperature for 3 h until gas formation ended and the solvent was removed. A mixture of valine methyl ester hydrochloride (0.066 g, 0.393 mmol, 1 equiv), CH_2Cl_2 (0.65 mL) and water (0.65 mL) was prepared. K_2CO_3 (0.163 g, 1.179 mmol, 3 equiv) was added to the resulting solution at 0 °C. The mixture was stirred at 0 °C for 5 min before the freshly synthesized (*Z*)-hexadec-9-enoyl chloride

in CH_2Cl_2 (0.65 mL) was added. After stirring at room temperature for 16 h the mixture was extracted with CH_2Cl_2 (3 × 10 mL), dried with $MgSO_4$, and concentrated in vacuo. Flash column chromatography (pentane/diethyl ether 1:1) provided the pure compound **9** as colorless oil (0.120 g, 0.326 mmol, 83%).

TLC [Pentane/diethyl ether (1:1)]: R_f = 0.31;

^{1}H-NMR ($CDCl_3$, 300 MHz): δ (ppm) = 6.00 (d, *J* = 8.7 Hz, 1 H), 5.35 (m, 2 H), 4.59 (dd, *J* = 8.8 Hz, *J* = 5.0 Hz, 1 H), 3.74 (s, 3 H), 2.23 (t, 2 H), 1.94-2.20 (m, 3 H),1.64 (m, 2 H), 1.31 (s, 24 H), 0.92 (m, 9 H);

^{13}C-NMR ($CDCl_3$, 100 MHz): δ (ppm) = 173.0 (NHC=O), 172.7 (C=O), 130.0 (CH), 129.7 (CH), 56.7 (CH), 52.0 (CH_3), 36.6 (CH_2), 31.7 (CH_2), 31.2 (CH), 29.7 (CH_2), 29.7 (CH_2), 29.3 (CH_2), 29.2 (CH_2), 29.1 (CH_2), 28.9 (CH_2), 27.2 (CH_2), 27.1 (CH_2), 25.6 (CH_2), 22.6 (CH_2), 18.9 (CH_3), 17.8 (CH_3), 14.0 (CH_3);

IR (diamond-ATR): $\tilde{\nu}$ = 3302 (w, br) cm^{-1}, 3003 (w), 2957 (m), 2925 (m), 2854 (m), 1745 (m), 1648 (s), 1535 (m), 1463 (w), 1436 (w), 1373 (w), 1309 (w), 1262 (w), 1203 (m), 1153 (m), 1118 (w), 1023 (w), 1004 (w), 919 (w), 730 (m).

EI-MS (70 eV): *m/z* (%) = 367 (5) [$M^{+\cdot}$], 308 (8), 292 (2), 254 (4), 237 (3), 206 (2), 186 (7), 173 (6), 154 (3), 132 (100), 114 (6), 98 (6), 88 (7), 72 (93), 67 (10), 55 (23), 41 (10), 39 (2).

Methyl *N*-hexadecanoylglycinate (10)

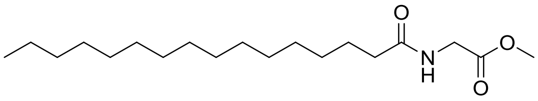

10

Compound **10** was prepared according to the procedure for **9** from palmitic acid (1.38 g, 5.37 mmol) glycine methyl ester hydrochloride (0.67 g, 5.37 mmol) as a white crystalline solid (1.48 g, 4.52 mmol, 84%).

TLC [Acetonitrile]: R_f = 0.38;

^{1}H-NMR ($CDCl_3$, 300 MHz, TMS): δ (ppm) = 5.99 (br. s, 1 H), 4.05 (d, J = 5.1 Hz, 2 H), 3.77 (s, 3 H), 2.24 (m, 2 H), 1.57 (m, 2 H), 1.17 (m, 24 H), 0.81 (m, 3 H);

^{13}C-NMR ($CDCl_3$, 100 MHz, TMS): δ (ppm) = 172.9 (NHC=O), 170.3 (C=O), 51.9 (CH_3), 410.8 (CH_2), 36.0 (CH_2C=O), 31.6 (CH_2), 29.3 (CH_2), 29.3 (CH_2), 29.2 (CH_2), 29.2 (CH_2), 29.1 (CH_2), 28.9 (CH_2), 28.8 (CH_2), 28.6 (CH_2), 25.2 (CH_2), 22.3 (CH_2), 13.7 (CH_3);

IR (diamond-ATR): $\tilde{\nu}$ = 3302 (w) cm^{-1}, 2957 (w), 2918 (s), 2849 (m), 1738 (m), 1639 (s), 1545 (m), 1460 (w), 1435 (m), 1375 (m), 1270 (w), 1214 (s), 1181 (m), 1124 (w), 1084 (w), 1044 (w), 991 (w), 890 (w), 872 (w), 718 (m), 678 (m), 607 (w);

EI-MS (70 eV): m/z (%) = 327 (2) [$M^{+\cdot}$], 239 (6), 167 (4), 149 (11), 144 (20), 132 (9), 131 (100), 112 (8), 103 (11), 99 (9), 90 (28), 89 (5), 69 (7), 57 (9), 55 (12), 43 (11), 41 (8).

Methyl (*Z*)-*N*-hexadec-9-enoylglycinate (11)

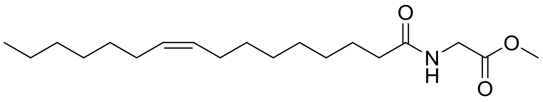

11

Compound **11** was prepared according to the procedure for **9** from palmitoleic acid (**S7**, 0.250 g, 0.98 mmol) and glycine methyl ester hydrochloride (0.123 g, 0.98 mmol, 1 equiv) as colorless oil (0.246 g, 0.76 mmol, 77%).

TLC [Pentane/diethyl ether (1:2)]: R_f = 0.33;

^{1}H-NMR ($CDCl_3$, 300 MHz, TMS): δ (ppm) = 6.13 (br. s, 1 H), 5.34 (m, 2 H), 4.05 (d, J = 5.2 Hz, 2 H), 3.76 (s, 3 H), 2.24 (m, 2 H), 2.01 (m, 4 H), 1.63 (dd, J = 14.6 Hz, J = 7.2 Hz, 2 H), 1.30 (m, 16 H), 0.88 (dd, J = 9.3 Hz , J = 4.2 Hz, 3 H);

^{13}C-NMR ($CDCl_3$, 100 MHz, TMS): δ (ppm) = 173.3 (NHC=O), 170.5 (C=O), 129.9 (CH), 129.7 (CH), 52.2 (CH_3), 41.1 (CH_2), 36.3 (CH_2), 31.7 (CH_2), 29.7 (CH_2), 29.6 (CH_2), 29.2 (CH_2), 29.1 (CH_2), 29.0 (CH_2), 28.9 (CH_2), 27.1 (CH_2), 27.0 (CH_2), 25.5 (CH_2), 22.6 (CH_2), 14.0 (CH_2);

IR (diamond-ATR): $\tilde{\nu}$ = 3301 (w, br) cm^{-1}, 3076 (w), 3004 (w), 2924 (m), 2854 (m), 1756 (m), 1652 (m), 1539 (m), 1460 (w), 1437 (w), 1370 (w), 1203 (s), 1180 (m), 1036 (w), 987 (w), 846 (w), 722 (m), 706 (m), 561 (w);

EI-MS (70 eV): *m/z* (%) = 325 (3) [$M^{+\cdot}$], 294 (3), 266 (4), 237 (2), 236 (3), 206 (3), 198 (3), 143 (12), 131 (26), 112 (6), 98 (11), 95 (9), 90 (100), 81 (11), 69 (10), 55 (18), 41 (9).

Methyl *N*-hexadecanoyl-2-aminobutyrate (7)

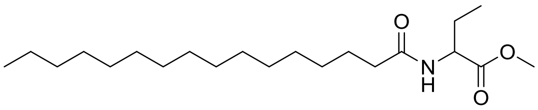

7

Compound **7** was prepared according to the procedure for **9** from palmitic acid (0.497 g, 1.94 mmol) and L-2-aminobutyric acid methyl ester hydrochloride (0.200 g, 1.94 mmol) as colorless oil (0.590 g, 1.66 mmol, 86%) which crystalized on standing.

TLC [Pentane/diethyl ether (1:1)]: R_f = 0.28;

^{1}H-NMR ($CDCl_3$, 300 MHz, TMS): δ (ppm) = 6.03 (d, *J* = 7.7 Hz, 1 H, NH), 4.60 (ddd, *J* = 7.9 Hz, *J* = 6.7 Hz, *J* = 5.5 Hz, 1 H, CH), 3.74 (s, 3 H, OCH_3), 2.20 (t, 2H, CH_3CO), 1.83-1.94 (m, 2 H, CH_2),1.58-1.76 (m, 2 H, CH_2), 1.25 (s, 24 H, CH_2), 0.90 (q, *J* = 7.2 Hz, 6 H, CH_3);

^{13}C-NMR ($CDCl_3$, 100 MHz, TMS): δ (ppm) = 173.1 (NHC=O), 172.8 (C=O), 53.0 (CH), 52.2 (CH_3), 36.6 (CH_2), 31.9 (CH_3), 29.65 (CH_2), 29.61 (CH_2), 29.57 (CH),

29.45 (CH_2), 29.32 (CH_2), 29.30 (CH_2), 29.21 (CH_2), 25.65 (CH_2), 25.63 (CH_2), 24.85 (CH_2), 22.65 (CH_2), 14.1 (CH_3), 9.4 (CH_3);

IR (diamond-ATR): $\tilde{\nu}$ = 3305 (w) cm^{-1}, 3076 (w), 2960 (m), 2917 (s), 2849 (m), 1741 (s), 1646 (s), 1548 (m), 1461 (w), 1435 (w), 1383 (w), 1313 (w), 1263 (3), 1246 (m), 1211 (m), 1152 (w), 1098 (w), 1070 (w), 1006 (m), 956 (w), 698 (m);

EI-MS (70 eV): *m/z* (%) = 355 (8) $[M]^{+\cdot}$, 324 (4), 312 (1), 296 (33), 256 (7), 239 (4), 228 (2), 214 (2), 196 (4), 182 (2), 172 (14), 159 (65), 140 (8), 131 (8), 118 (21), 100 (19), 83 (7), 69 (10), 58 (100), 43 (22).

Methyl (*Z*)-hexadec-9-enoyl-2-aminobutyrate (8)

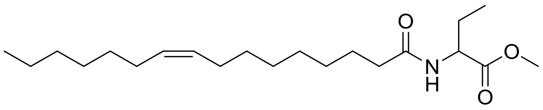

8

Compound **8** was prepared according to the procedure for **9** from palmitoleic acid (**S7**, 0.984 g, 3.79 mmol) and L-2-aminobutyric acid methyl ester hydrochloride (0.400 g, 3.88 mmol) as yellowish oil (0.955 g, 2.70 mmol, 70%).

TLC [Pentane/diethyl ether (1.5:1)]: R_f = 0.27;

^{1}H-NMR ($CDCl_3$, 300 MHz, TMS): δ (ppm) = 5.99 (d, *J* = 7.1 Hz, 1 H, NH), 5.34 (m, 2 H), 4.6 (m, 1 H), 3.7 (s, 3 H), 2.23 (m, 2 H), 1.92-2.30 (m, 4 H), 1.58-1.79 (m, 4 H), 1.24-1.38 (m, 16 H), 0.90 (m, 6 H);

^{13}C-NMR ($CDCl_3$, 100 MHz, TMS): δ (ppm) = 172.7 (NHC=O), 172.4 (C=O), 129.5 (CH), 129.3 (CH), 52.6 (CH), 51.8 (CH_3), 36.2 (CH_2), 31.3 (CH_2), 29.3 (CH_2), 29.2 (CH_2), 28.8 (CH_2), 28.8 (CH_2), 28.7 (CH_2), 28.5 (CH_2), 26.8 (CH_2), 26.7 (CH_2), 25.2 (CH_2), 25.2 (CH_2), 22.2 (CH_2), 13.6 (CH_3), 9.0 (CH_3);

IR (diamond-ATR): $\tilde{\nu}$ = 3298 (w, br) cm^{-1}, 3063 (w), 2926 (m), 2855 (m), 1743 (s), 1649 (s), 1536 (m), 1459 (m), 1438 (w), 1374 (w), 1295 (w), 1255 (m), 1203 (s), 1155 (m), 988 (w), 725 (w), 680 (w);

EI-MS (70 eV): *m/z* (%) = 353 (16) [M]$^{+\cdot}$, 322 (3), 310 (1), 294 (12), 264 (2), 254 (2), 236 (4), 226 (4), 214 (2), 193 (2), 186 (1), 172 (16), 159 (16), 140 (6), 131 (3), 118 (100), 100 (8), 95 (7), 81 (10), 69 (12), 58 (57), 41 (11).

Synthesis of palmitoleic acid (S7)

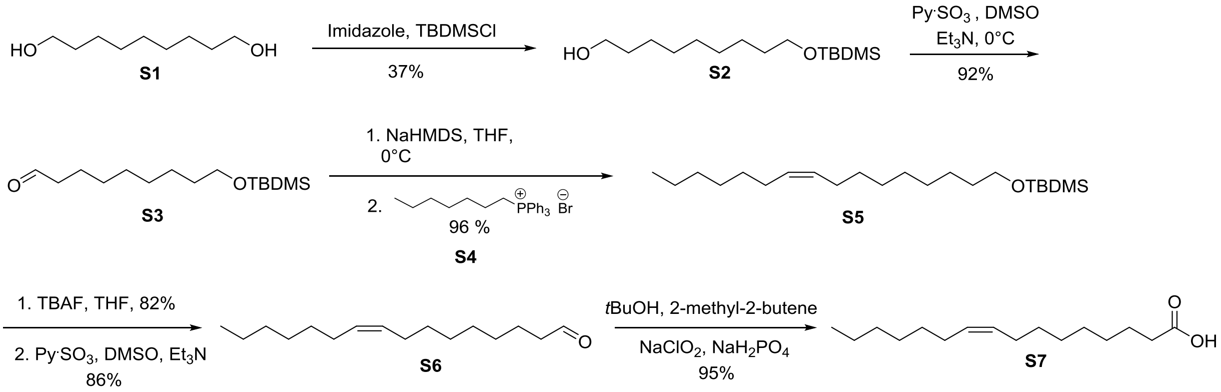

Scheme S1: Synthesis of palmitoleic acid in g-scale [S8].

9-((*tert*-Butyldimethylsilyl)oxy)nonan-1-ol (S2)

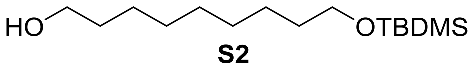

S2

In a similar manner as described in [S1], a solution of 1,9-nonandiol (**S1**, 8.013 g, 50 mmol, 1 equiv) in dry DCM (450 mL) imidazole (4.425 g, 65 mmol, 1.3 equiv) was added at 0 °C and stirred for 15 min, followed by addition of TBDMSCl (9.796 g, 65 mmol, 1.3 equiv) in portions. The solution was poured into H_2O after stirring at rt for 2.5 days, and extracted with ethyl acetate (3 × 100 mL). The combined organic phases were washed with brine, dried with Na_2SO_4, filtered, and concentrated in

vacuo. Chromatographic separation on silica with pentane/EtOAc (5:1) gave **S2** (5.050 g, 18.4 mmol, 37%).

TLC [Pentane/ethyl acetate (2:1)]: R_f = 0.33;

^{1}H-NMR ($CDCl_3$, 300 MHz): δ (ppm) = 4.1 (t, 1 H), 3.62 (td, J = 6.6 Hz, J = 13.2 Hz, 4 H), 1.53 (m, 4 H), 1.32 (m, 10 H), 0.89 (s, 9 H), –0.052 (s, 6 H);

^{13}C-NMR ($CDCl_3$, 100 MHz): δ (ppm) = 63.4 (CH_2OH), 63.1 (CH_2), 32.9 (CH_2), 32.9 (CH_2), 29.6 (CH_2), 29.4 (2 C, CH_2), 26.0 (3 C, CH_3), 25.8 (CH_2), 25.8 (CH_2), 18.4 (C_q), -5.2 (2 C, CH_3);

EI-MS (70 eV): *m/z* (%) = 217 (2), 199 (3), 115 (10), 105 (37), 101 (12), 99 (6), 93 (17), 89 (10), 83 (66), 75 (80), 73 (25), 69 (100), 55 (57), 41 (20).

9-(*tert*-Butyldimethylsilyloxy)nonanal (S3)

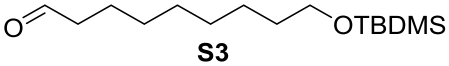

S3

In a similar manner as described in [S1], a solution of $SO_3 \cdot py$ (8.7 g, 54.6 mmol, 3 equiv) in DMSO (56 mL) was added dropwise to a solution of **S2** (5.00 g, 18.2 mmol, 1 equiv) and Et_3N (30.4 g, 300 mmol, 16.5 equiv) in dry DCM (162 mL) at 0 °C under argon. The resulting mixture was allowed to stir at rt for 2.5 h until the reaction was completed. The reaction was quenched with H_2O, extracted with DCM (3 × 100 mL), washed with brine (3 × 60 mL), dried with $MgSO_4$, and concentrated in vacuo to afford the crude product. Flash column chromatography (pentane/Et_2O (19:1)) gave the pure title compound **S3** as clear oil (4.836 g, 17.7 mmol, 92%).

TLC [Pentane/diethyl ether (19:1)]: R_f = 0.31;

^{1}H-NMR ($CDCl_3$, 300 MHz): δ (ppm) = 9.76 (t, CHO, 1 H), 3.59 (t, J = 4.8 Hz, 2 H), 2.42 (dt, J = 1.9 Hz, J = 7.3 Hz, 2 H), 1.57 (m, 4 H), 1.30 (s, 8 H), 0.89 (m, 9 H), 0.046 (s, 6 H);

^{13}C-NMR ($CDCl_3$, 100 MHz): δ (ppm) = 202.9 (*C*HO), 63.2 (CH_2), 43.9 (CH_2), 32.8 (CH_2), 29.4 (CH_2), 29.3 (CH_2), 29.2 (CH_2), 29.1 (CH_2), 26.0 (3 C, CH_3), 22.1 (CH_2), 18.4 (CH_2), –5.3 (2 C, CH_3);

IR (diamond-ATR): $\tilde{\nu}$ = 2929 (m) cm^{-1}, 2856 (m), 2712 (w), 1727 (m), 1466 (w), 1388 (w), 1361 (w), 1253 (w), 1095 (m), 1006 (w), 938 (w), 833 (s), 774 (s), 713 (w), 662 (w);

EI-MS (70 eV): *m/z* (%) = 215 (9), 131 (30), 123 (6), 115 (6), 105 (24), 101 (9), 89 (11), 81 (75), 75 (100), 73 (22), 67 (33), 55 (16), 41 (10).

(*Z*)-*tert*-Butyl(hexadec-9-en-1-yloxy)dimethylsilane (S5)

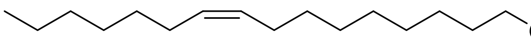

S5

NaHMDS (1.0 M in THF, 38.51 mL, 38.51 mmol, 2.1 equiv) was added dropwise to a stirred solution of the hexyl triphenylphosphonium bromide **S4** (8.50 g, 19.26 mmol, 1.05 equiv) in THF (68 mL) at 0 °C. The reaction mixture was stirred at rt for 45 min. The bright orange solution was recooled to −78 °C before **S3** (5 g, 18.34 mmol, 1 equiv) was added. After stirring for 1 h at −78 °C, the mixture was allowed to warm to rt and poured into ice-cooled pentane. Ph_3PO was filtered off and two third of the solvent was evaporated under reduced pressure. The mixture was adsorbed on SiO_2 and purification of the crude residue by column chromatography (pentane/Et_2O (80:1)) gave **S5** (6.56 g, 18.49 mmol, 96%) as clear, colorless oil.

TLC [Pentane/diethyl ether (80:1)]: R_f = 0.33;

^{1}H-NMR ($CDCl_3$, 300 MHz): δ (ppm) = 5.35 (m, 2 H), 3.59 (t, 2 H), 2.01 (m, 4 H), 1.52 (m, 2 H), 1.28 (m, 18 H), 0.89 (m, 12 H), 0.046 (s, 6 H);

^{13}C-NMR ($CDCl_3$, 100 MHz): δ (ppm) = 129.9 (CH), 129.9 (CH), 63.3 (CH_2), 32.9 (CH_2), 31.8 (CH_2), 29.8 (2 C, CH_2), 29.5 (CH_2), 29.4 (CH_2), 29.3 (CH_2), 29.0 (CH_2),

27.2 (CH_2), 27.2 (CH_2), 26.0 (3 C, CH_3), 25.8 (CH_2), 22.7 (CH_2), 18.4 (C-*t*Bu), 14.1 (CH_3), –5.3 (2 C, CH_3);

IR (Diamond-ATR): $\tilde{\nu}$ = 3005 (w) cm^{-1}, 2925 (m), 2855 (m), 1464 (w), 1386 (w), 1253 (w), 1098 (m), 1006 (w), 834 (s), 774 (m), 723 (w), 661 (w), 542 (w);

EI-MS (70 eV): *m/z* (%) = 339 (3), 299 (11), 298 (37), 297 (100), 269 (34), 115 (10), 109 (15), 101 (12), 95 (21), 89 (19), 75 (91), 67 (13), 55 (19), 41 (13).

(*Z*)-Hexadec-9-en-1-ol

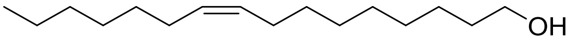

Aldehyde **S5** (7.15 g, 20.15 mmol, 1 equiv) was added to a THF solution (76 mL) of TBAF (1 M in THF, 48.38 mL, 48.38 mmol, 2.4 equiv) at 0 °C. The resulting mixture was stirred at 0 °C for 5 min and at rt for 3 h until the reaction was completed. The solution was quenched with H_2O, extracted with Et_2O (3 × 50 mL), washed with brine, dried with Na_2SO_4 and concentrated under reduced pressure. The residue was purified by flash chromatography (pentane/ethyl acetate (5:1)) affording (*Z*)-hexadec-9-en-1-ol (3.96 g, 16.44 mmol, 82%) as clear, colorless oil.

TLC [Pentane/ethyl acetate (5:1)]: R_f = 0.36;

^{1}H-NMR ($CDCl_3$, 300 MHz): δ (ppm) = 6.34 (m, 2 H), 3.63 (t, 2 H), 2.02 (m, 4 H), 1.56 (m, 2 H), 1.29 (m, 18 H), 0.88 (t, 3 H);

^{13}C-NMR ($CDCl_3$, 100 MHz): δ (ppm) = 129.9 (CH), 129.8 (CH), 63.0 (CH_2), 32.8 (CH_2), 31.8 (CH_2), 29.7 (CH_2), 29.5 (CH_2), 29.4 (CH_2), 29.2 (CH_2), 29.0 (CH_2), 27.2 (CH_2), 27.2 (CH_2), 25.7 (CH_2), 22.6 (CH_2), 14.1 (CH_3);

IR (diamond-ATR): $\tilde{\nu}$ = 3328 (br, w) cm^{-1}, 3005 (w), 2923 (s), 2854 (m), 1462 (w), 1378 (w), 1055 (w), 883 (w), 722 (w);

EI-MS (70 eV): *m/z* (%) = 222 (17), 138 (12), 123 (24), 109 (44), 96 (83), 82 (100), 67 (76), 55 (88), 41 (52).

(*Z*)-Hexadec-9-enal (S6)

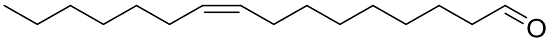

S6

The oxidation was performed as described for **S3** from (*Z*)-hexadec-9-en-1-ol (4.11 g, 17.09 mmol) to afford by flash column chromatography (pentane/Et_2O (19:1)) the pure **S6** as clear oil (3.48 g, 14.62 mmol, 86%).

TLC [Pentane/diethyl ether (19:1)]: R_f = 0.32;

^{1}H-NMR ($CDCl_3$, 300 MHz): δ (ppm) = 9.68 (t, *J* = 1.88 Hz, 1 H), 5.25 (m, 2 H), 2.34 (td, 2 H), 1.92 (m, 4 H), 1.53 (m, 2 H), 1.22 (m, 16 H), 0.82 (t, 3 H);

^{13}C-NMR ($CDCl_3$, 100 MHz): δ (ppm) = 202.9 (*C*HO), 130.0 (CH), 129.7 (CH), 43.9 (CH_2), 31.7 (CH_2), 29.7 (CH_2), 29.6 (CH_2), 29.2 (CH_2), 29.1 (CH_2), 29.0 (CH_2), 29.0 (CH_2), 27.2 (CH_2), 25.6 (CH_2), 22.6 (CH_2), 22.0 (CH_2), 14.1 (CH_3);

EI-MS (70 eV): *m/z* (%) = 238 (3) [M^+], 220 (9), 163 (3), 149 (6), 138 (7), 135 (17), 121 (30), 111 (29), 98 (55), 81 (63), 69 (73), 55 (100), 41 (71), 39 (14).

(*Z*)-Hexadec-9-enoic acid (S7)

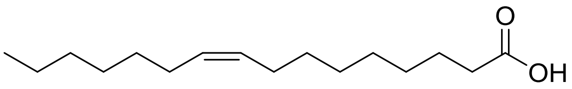

S7

A solution of $NaClO_2$ (9.26 g, 102 mmol, 7 equiv) and $NaH_2PO_4 \cdot H_2O$ (18.12 g, 132 mmol, 9 equiv) in H_2O (120 mL) was added dropwise into a mixture of aldehyde **S6** (3.48 g, 14.6 mmol, 1 equiv) and 2-methyl-2-butene (60 mL) in *t*-BuOH/THF (359 mL and 298 mL, respectively). The resulting mixture was stirred at rt until the reaction was completed. The reaction was quenched with brine and extracted with DCM. The

organic phase was washed with brine (3 × 150 mL), dried with $MgSO_4$, and concentrated in vacuo to afford the crude product. Flash column chromatography (pentane/ethyl acetate (10:1)) gave the pure title compound **S7** as clear colorless oil (3.53 g, 13.9 mmol, 95%).

TLC [Pentane/ethyl acetate (10:1)]: R_f = 0.37;

^{1}H-NMR ($CDCl_3$, 300 MHz): δ (ppm) = 6.13 (br.s, 1 H), 5.25 (m, 2 H), 2.24 (t, J = 7.50 Hz, 2 H), 1.91 (m, 4 H), 1.41 (m, 2 H), 1.19 (m, 16 H), 0.88 (t, 3 H);

^{13}C-NMR ($CDCl_3$, 100 MHz): δ (ppm) = 179.5 (C=O), 130.0 (CH), 129.7 (CH), 31.8 (CH_2), 29.7 (CH_2), 29.7 (CH_2), 29.1 (CH_2), 29.0 (CH_2), 29.0 (CH_2), 29.0 (CH_2), 27.2 (CH_2), 27.1 (CH_2), 25.6 (CH_2), 24.7 (CH_2), 22.6 (CH_2), 14.1 (CH_3);

EI-MS (70 eV) of trimethylsilyl ester: *m/z* (%) = 326 (7) [M^+], 311 (100), 236 (9), 199 (10), 194 (16), 185 (10), 152 (11), 145 (32), 129 (72), 117 (76), 96 (19), 81 (17), 75 (68), 55 (28), 41 (17).

NMR spectra

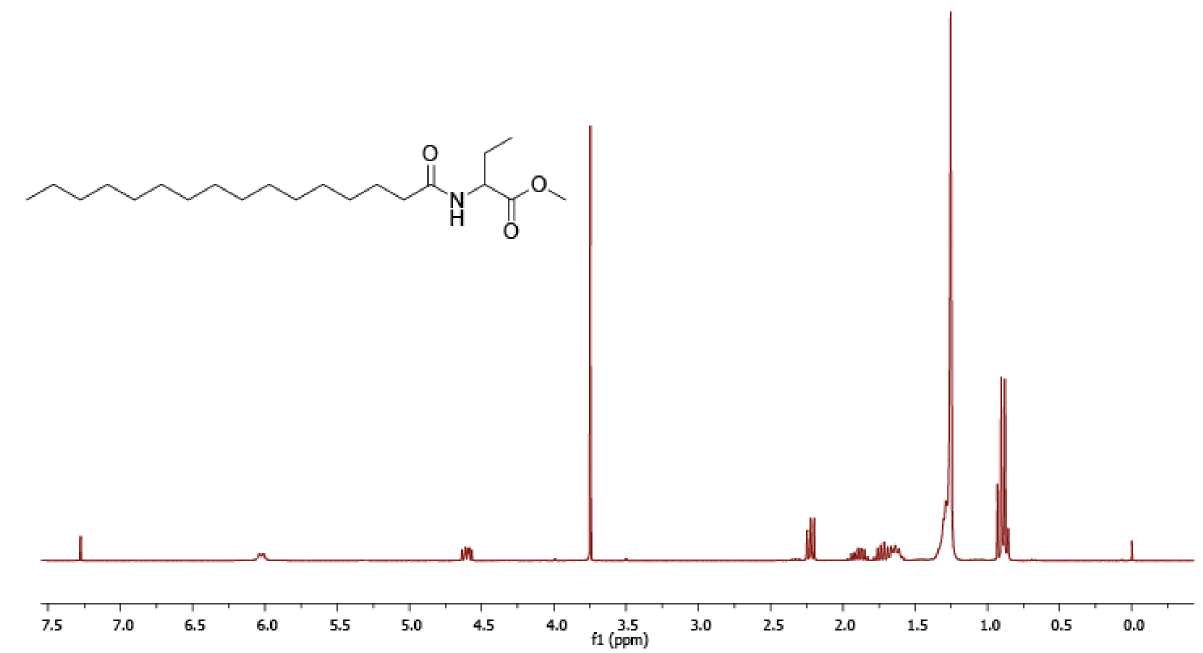

Figure S1: ^{1}H NMR spectrum of **7** (400 MHz, $CDCl_3$).

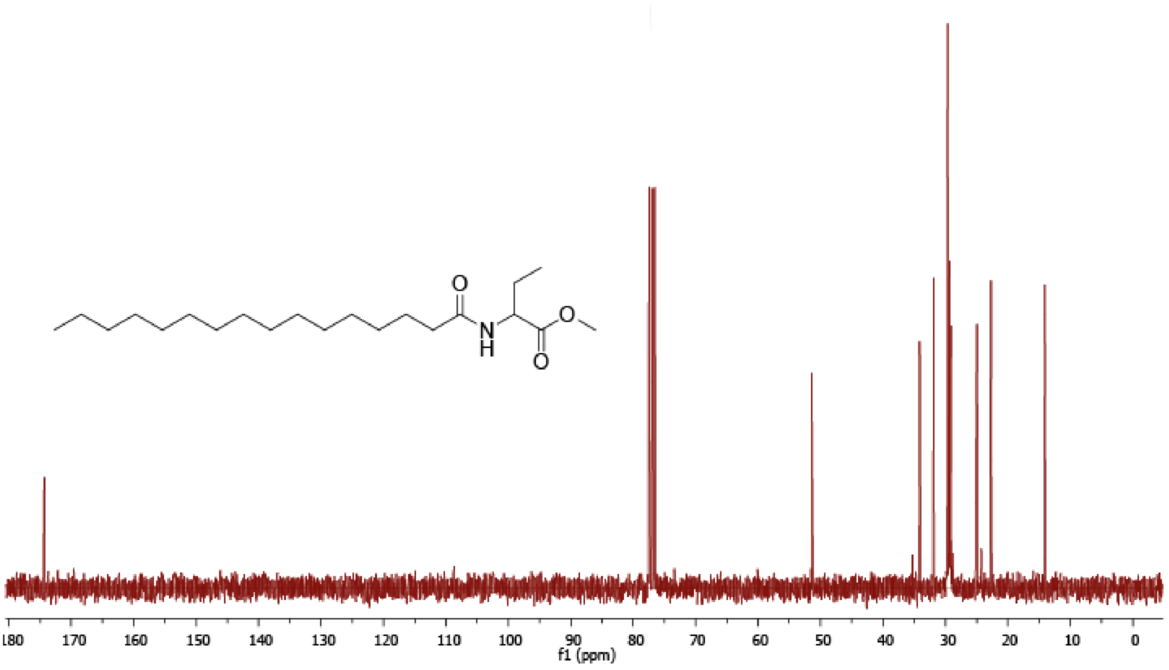

Figure S2: ^{13}C NMR spectrum of **7** (100 MHz, $CDCl_3$).

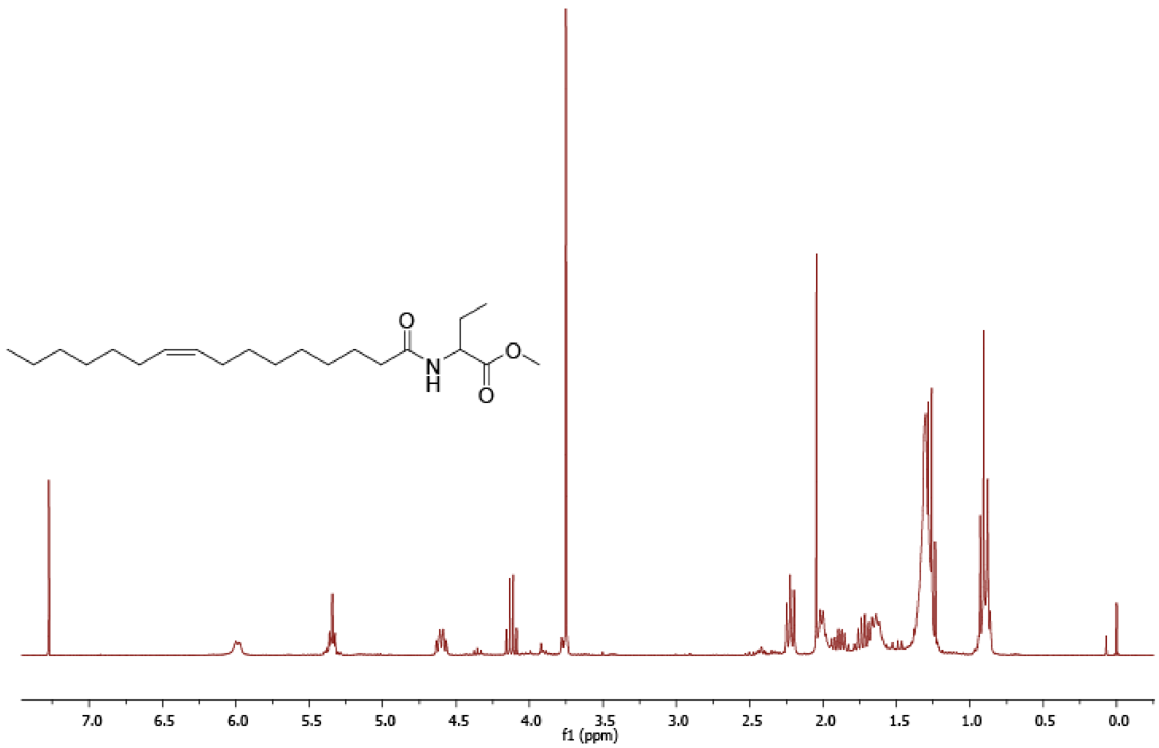

Figure S3: ^{1}H NMR spectrum of **8** (400 MHz, $CDCl_3$).

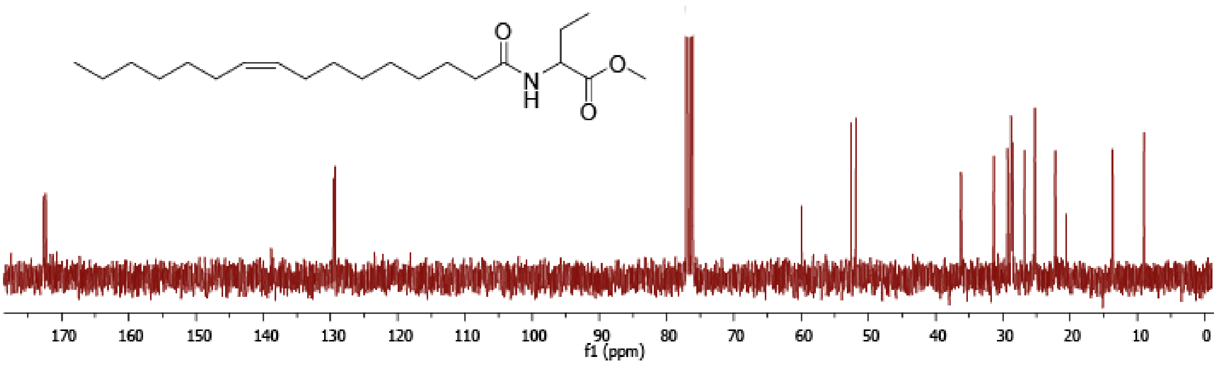

Figure S4: ^{13}C NMR spectrum of **8** (100 MHz, $CDCl_3$).

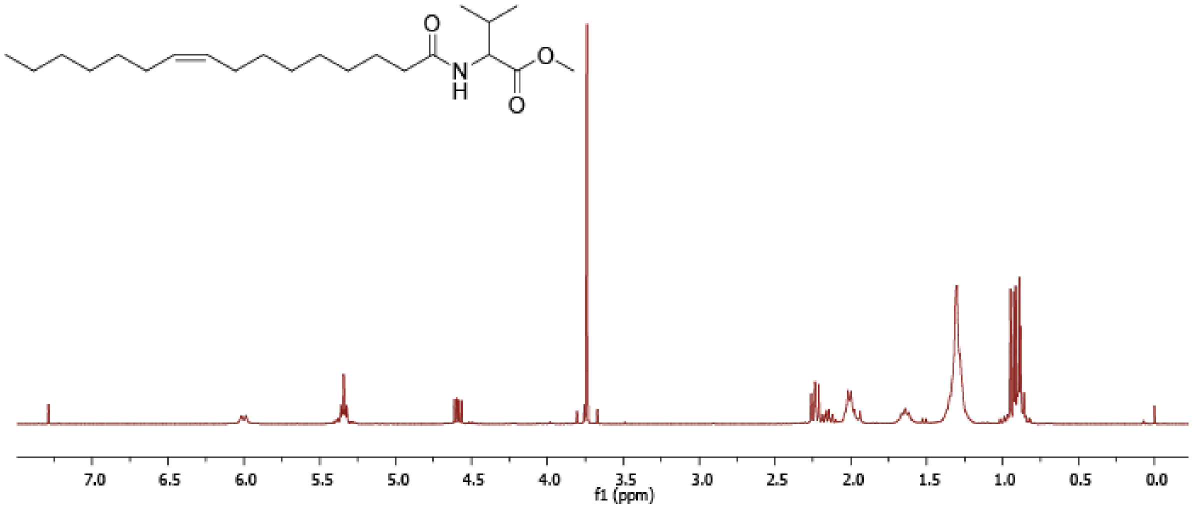

Figure S5: ^{1}H NMR spectrum of **9** (400 MHz, $CDCl_3$).

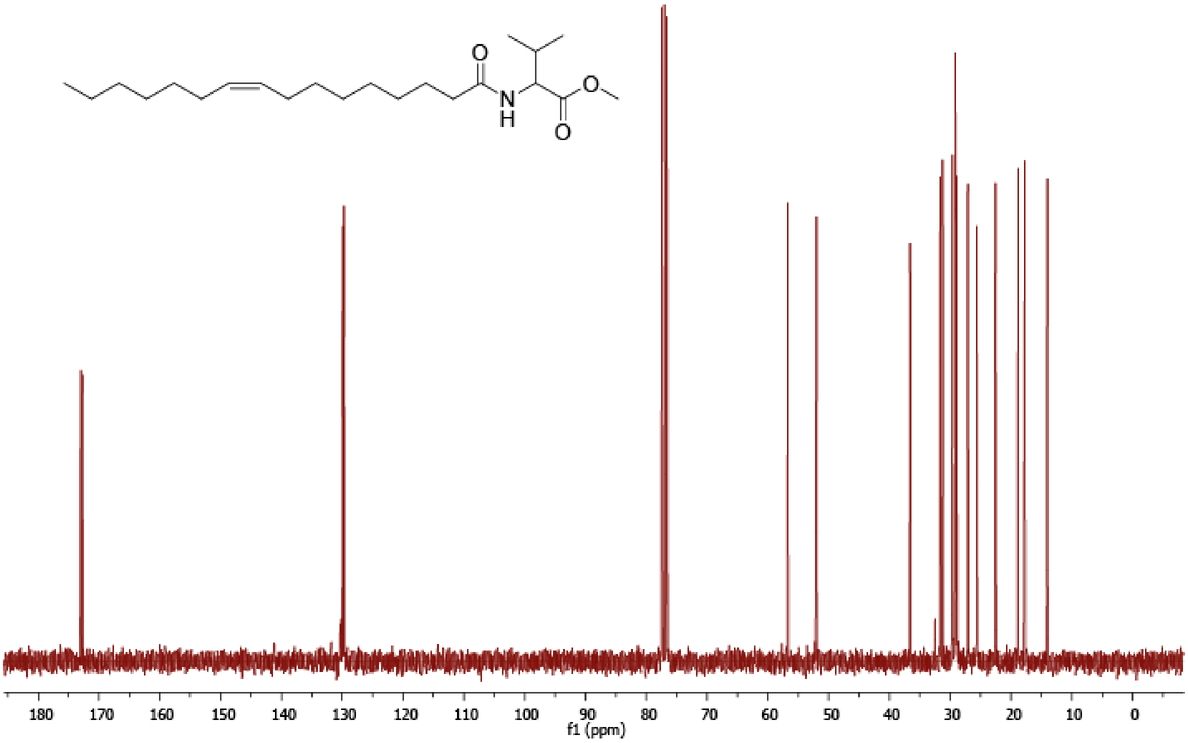

Figure S6: ^{13}C NMR spectrum of **9** (100 MHz, $CDCl_3$).

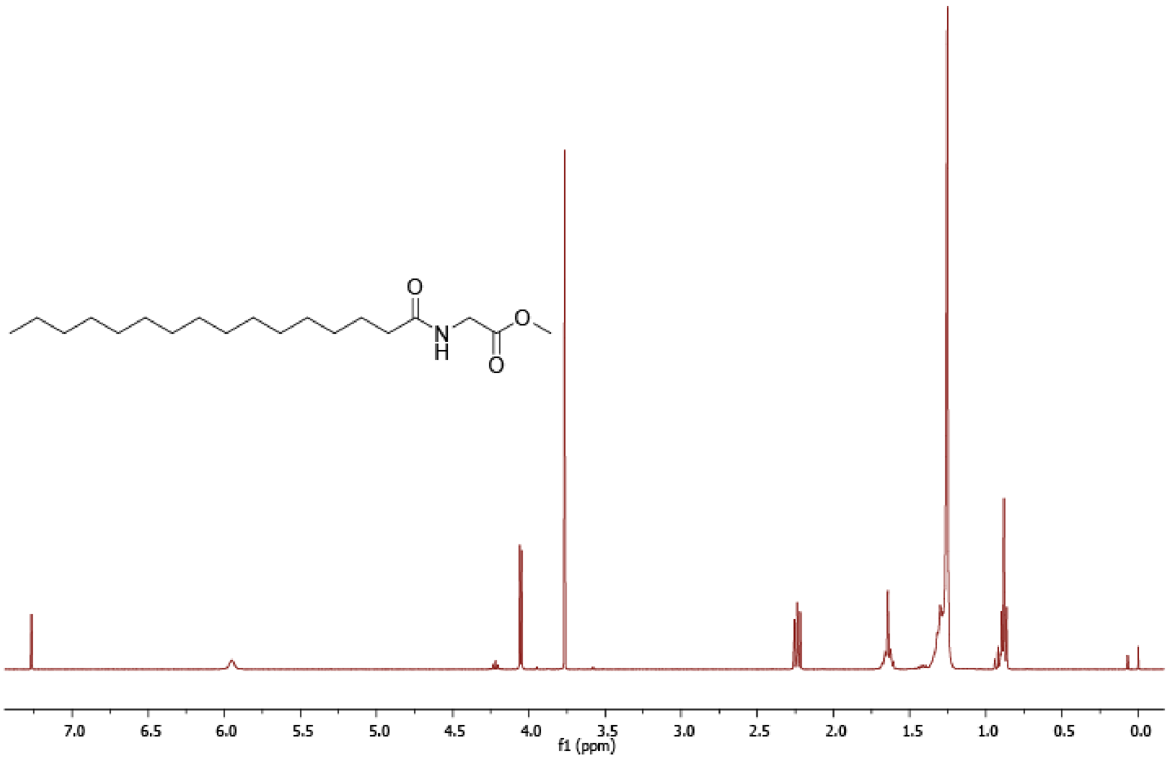

Figure S7: ^{1}H NMR spectrum of **10** (400 MHz, $CDCl_3$).

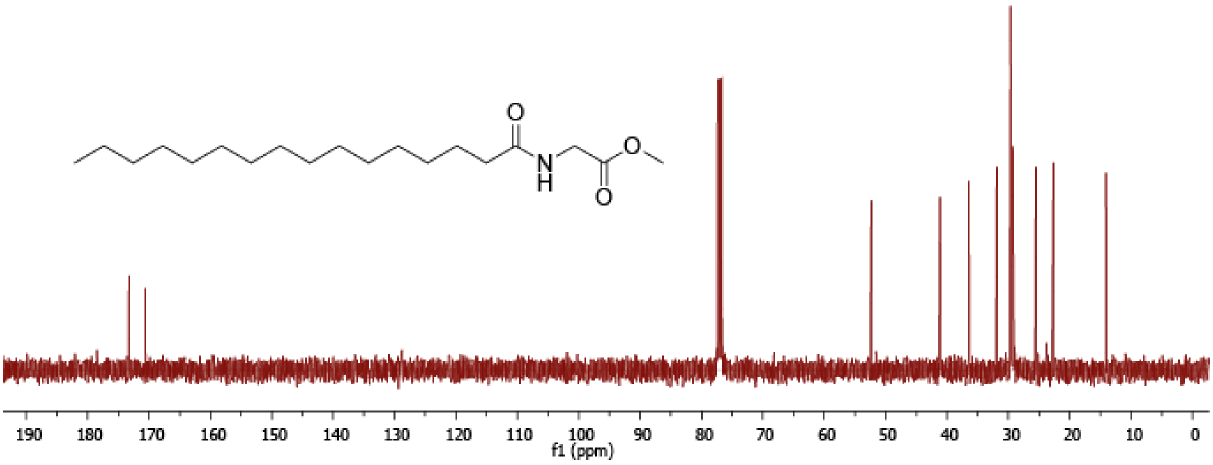

Figure S8: ^{13}C NMR spectrum of **10** (100 MHz, $CDCl_3$).

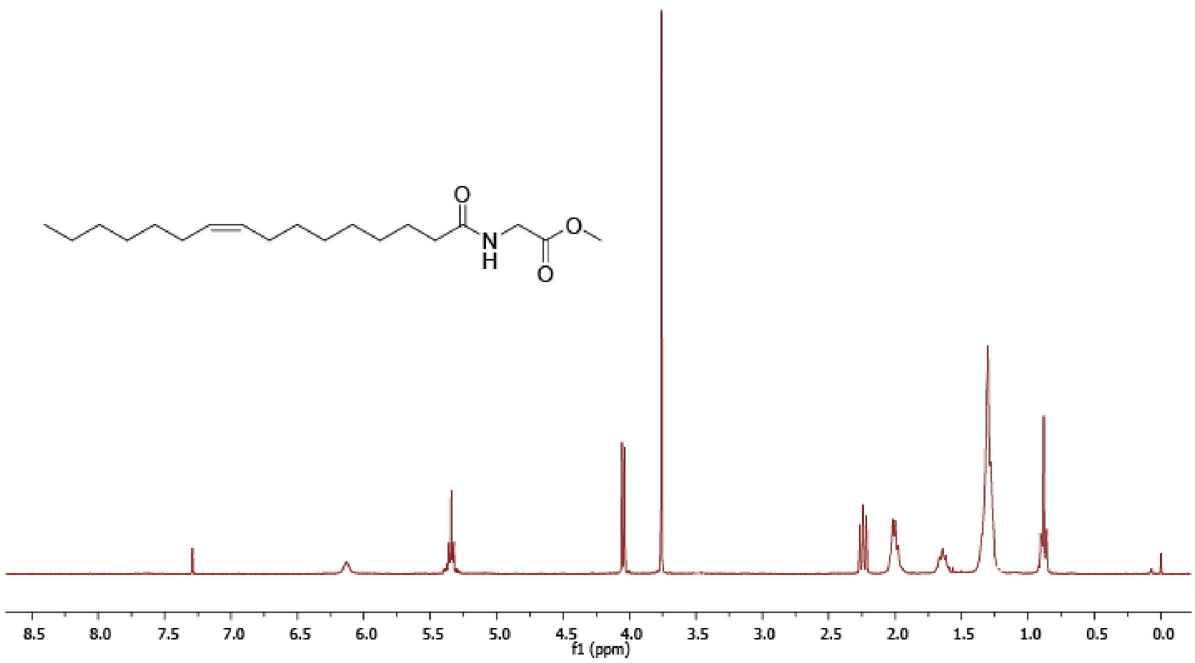

Figure S9: ^{1}H NMR spectrum of **11** (400 MHz, $CDCl_3$).

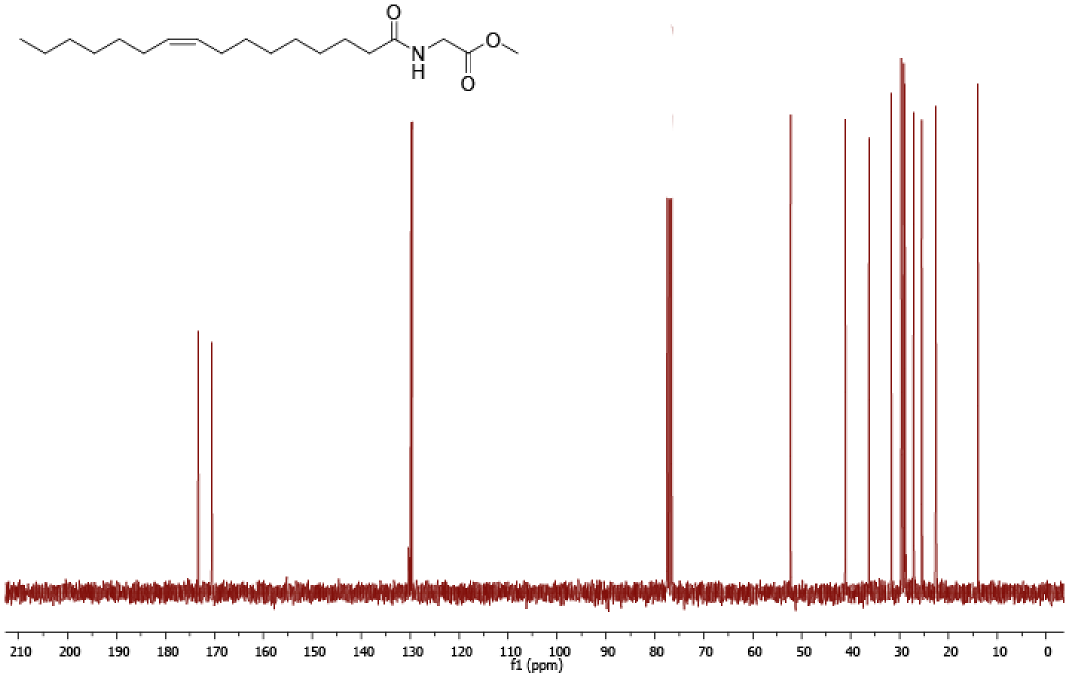

Figure S10: ^{13}C NMR spectrum of **11** (100 MHz, $CDCl_3$).

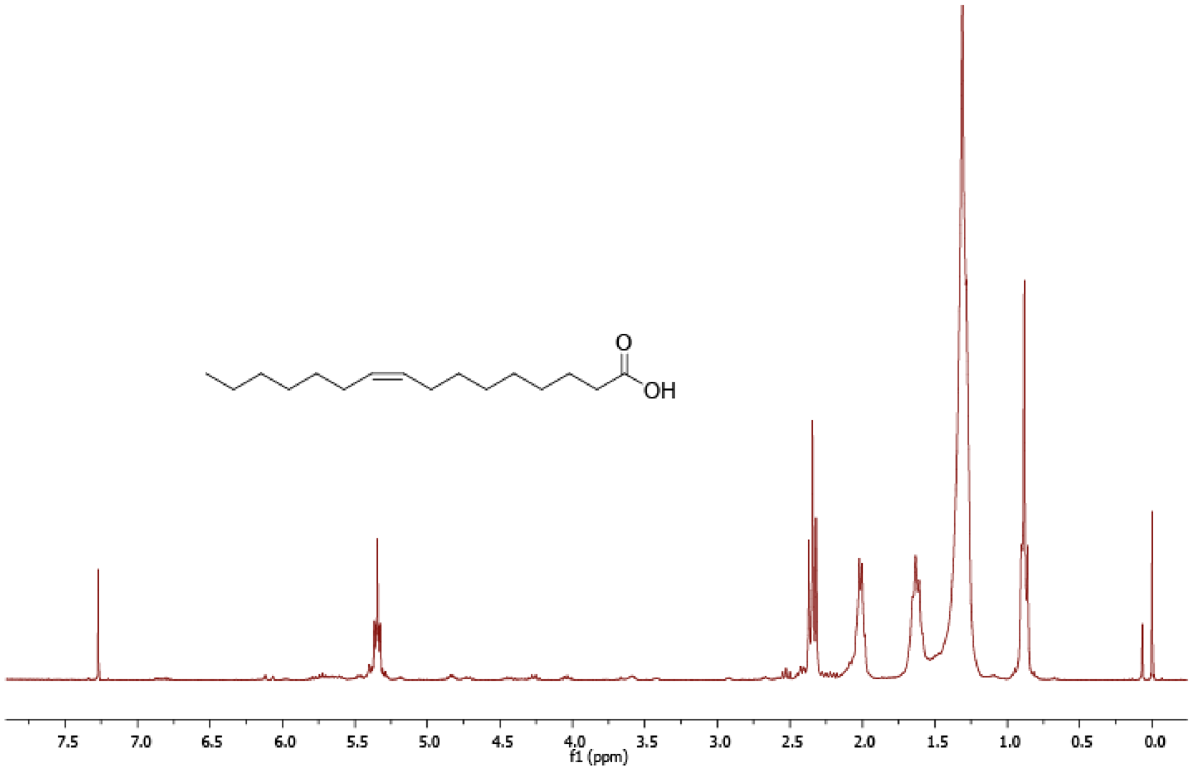

Figure S11: ^{1}H NMR spectrum of **S7** (400 MHz, $CDCl_3$).

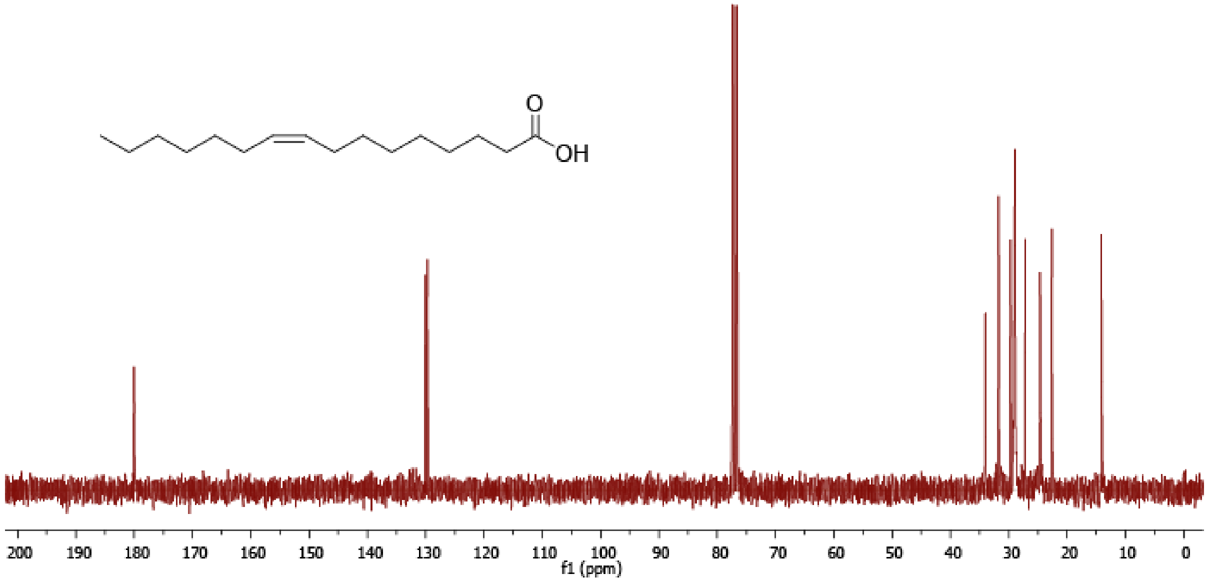

Figure S12: ^{13}C NMR spectrum of **S7** (100 MHz, $CDCl_3$).

References

S1. Bruns, H.; Herrmann, J.; Müller, R.; Wang, H.; Wagner Döbler, I.; Schulz, S. *J. Nat. Prod.* **2018,** *81*, 131–139. doi:10.1021/acs.jnatprod.7b00757

S2. Montes Vidal, D.; Rymon-Lipinski, A.-L. von; Ravella, S.; Groenhagen, U.; Herrmann, J.; Zaburannyi, N.; Zarbin, P. H. G.; Varadarajan, A. R.; Ahrens, C. H.; Weisskopf, L.; Müller, R.; Schulz, S. *Angew. Chem. Int. Ed.* **2017,** *56*, 4342–4346. doi:10.1002/anie.201611940

S3. Takikawa, H.; Nozawa, D.; Kayo, A.; Muto, S.-e.; Mori, K. *J. Chem. Soc., Perkin Trans. 1* **1999**, 2467–2477. doi:10.1039/A904258J

S4. Millar, J. G.; Oehlschlager, A. C.; Won, J. W. *J. Org. Chem.* **1983,** *48*, 4404–4407.

S5. Bowden, K.; Heilbron, I. M.; Jones, E. H.; Weedo, B. C. L. *J. Chem. Soc.* **1946,** *39*.

S6. Patel, J.; Hoyt, J. C.; Parry, R. J. *Tetrahedron* **1998,** *54*, 15927–15936. doi:10.1016/S0040-4020(98)01002-3

S7. Camarero, J. A.; Hackel, B. J.; Yoreo, J. J. de; Mitchell, A. R. *J. Org. Chem.* **2004,** *69*, 4145–4151. doi:10.1021/jo040140h

S8. Bruns, H.; Thiel, V.; Voget, S.; Patzelt, D.; Daniel, R.; Wagner-Döbler, I.; Schulz, S. *Chem. Biodiversity* **2013,** *10*, 1559–1573. doi:10.1002/cbdv.201300210
